# Positive association of unhealthy plant-based diets with the incidence of abdominal obesity in Korea: a comparison of baseline, most recent, and cumulative average diets

**DOI:** 10.4178/epih.e2022063

**Published:** 2022-08-02

**Authors:** Sukyoung Jung, Sohyun Park

**Affiliations:** 1 Department of Epidemiology, Milken Institute School of Public Health, George Washington University, Washington, DC, USA; 2 The Korean Institute of Nutrition, Hallym University, Chuncheon, Korea; 3 Department of Food Science and Nutrition, Hallym University, Chuncheon, Korea

**Keywords:** Plant-based diet index, Unhealthy plant foods, Abdominal obesity, Waist circumference, Prospective studies

## Abstract

**OBJECTIVES:**

Different approaches for analyzing repeated dietary measurements may yield differences in the magnitude and interpretation of findings. We aimed to compare 3 dietary measurements (baseline, most recent, and cumulative average) in terms of the association between plant-based diet indices (PDIs) and incident abdominal obesity in Korean adults aged 40-69 years.

**METHODS:**

This study included 6,054 participants (54% women) free of abdominal obesity (defined as waist circumference ≥90 cm for men and ≥85 cm for women) at baseline. As exposures, baseline, most recent, and cumulative average measurements for PDI, healthy-PDI (hPDI), and unhealthy-PDI (uPDI) were created. A Cox proportional-hazard model was used to estimate the hazard ratios (HRs) for abdominal obesity.

**RESULTS:**

During 45,818 person-years of follow-up (median, 9 years), we identified 1,778 incident cases of abdominal obesity. In the multivariable-adjusted analysis, a higher uPDI was associated with a higher risk of abdominal obesity in both total and stratified analyses. The findings were consistent across all approaches (Q5 vs. Q1: HR_baseline_=1.70; 95% confidence interval [CI], 1.46 to 1.98; HR_most recent_=1.52; 95% CI, 1.30 to 1.78; HR_cumulative average_=1.76; 95% CI, 1.51 to 2.06 in the total set). PDI showed no meaningful association with abdominal obesity risk in any analyses. HR_average_ had a suggestive inverse association with abdominal obesity risk in men, and hPDI_baseline_ had a positive association with abdominal obesity risk in women.

**CONCLUSIONS:**

Greater adherence to unhealthy plant-based diets may increase the risk of developing abdominal obesity in Korean adults. The findings were generally consistent across all approaches.

## INTRODUCTION

In recent years, plant-based diets have received extensive attention due to their reported benefits for both individual health and environmental sustainability [1], and previous meta-analyses have reported favorable effects of several types of plant-based diets [2,3] or foods [4-6] in relation to adiposity. However, some plant foods, such as refined grains, sugary beverages, and salted vegetables, are not considered healthy. In this context, 3 recently developed plant-based diet indices (PDIs), which consider less healthy plant foods and animal foods, can holistically assess not only the degree of adherence to overall plant-based diets, but also the quality of plant-based diets [7].

Visceral adiposity has long been recognized as a strong predictor of premature cardiometabolic diseases and death [8,9]. Waist circumference (WC) alone has been shown to have a stronger association with visceral fat than other non-imaging-based clinical methods such as the body mass index (BMI), waist-to-hip ratio, or waist-to-height ratio [8]. Furthermore, current evidence suggests that WC has an independent effect on cardiometabolic diseases and mortality [10-12]. Reflecting globally increasing trends over the past few decades [13], the prevalence of abdominal obesity assessed by WC among Korean adults aged 20 years and older increased from 2009 to 2018 (19.0% in 2009 to 23.8% in 2018) [14].

In previous observational studies focusing on adiposity as an outcome, the overall PDI showed inverse associations with weight gain [15], and the healthy PDI (hPDI), characterized by a high intake of whole grains, fruits, vegetables, nuts, legumes, tea, and coffee, showed inverse associations with adiposity-related inflammatory markers [16], visceral and subcutaneous abdominal adipose tissue volume [17], obesity defined by the BMI [18], and weight, WC, and the waist-to-hip ratio [15,19]. In contrast, the unhealthy PDI (uPDI), characterized by a high intake of refined grains, sweets, desserts, and sugary beverages, has shown positive associations with weight gain [15], metabolic syndrome [20,21], BMI, and WC [22]. However, the previous evidence has some limitations, including cross-sectional study designs [17,19,21,22]; inapplicability to different ages, genders, races/ethnicities, and socioeconomic statuses [15-18]; and relatively small sample sizes [17-19,22]. Furthermore, the cohort study design allows us to collect repeated dietary measurements over time. The use of repeated measurements provides an opportunity to: (1) consider possible changes in the dietary habits of participants during the follow-up period; (2) reduce measurement error; and (3) examine the different effects of several temporal assumptions in exposures (long-term vs. short-term diet intake) on outcomes [23]. However, no study has yet examined the possible differences in associations between plant-based diets and abdominal obesity risk by comparing several approaches for analyzing repeated dietary measurements.

We therefore aimed to compare 3 different approaches for analyzing repeated dietary measurements (baseline, most recent, and cumulative average diets) in the associations between 3 PDI scores (PDI, hPDI, and uPDI) and incident abdominal obesity among Korean adults aged 40-69 years.

## MATERIALS AND METHODS

### Study population

The Korean Genome and Epidemiology Study (KoGES) Ansan and Ansung Study, an ongoing population-based study, was established to determine risk factors for common complex diseases and death in Koreans [24]. A total of 10,030 participants aged 40 years to 69 years living in rural and urban communities in Korea were recruited between 2001 and 2002 through 2-stage cluster sampling: Ansan (an urban community) and Ansung (a rural community) [24]. The enrolled participants completed biennial follow-up examinations, starting in 2001-2002, and the follow-up rate was 62.8% in 2015-2016 at the 6th follow-up [24].

Among 7,056 participants who had a baseline WC < 90 cm for men and < 85 cm for women, we excluded those who had the following conditions at baseline: implausible (< 800 or > 4,000 kcal/day for men and < 500 or > 3,500 kcal/day for women) [23] or incomplete dietary intake (n=387); a history of heart disease, stroke, and/or cancer (n= 318); and missing data on key covariates (age, gender, total energy intake, education level, physical activity level, smoking status, alcohol intake, and BMI) (n= 297). The final analytic sample included 6,054 participants (3,273 men and 2,781 women) (Supplementary Material 1).

### Dietary intake and plant-based diet index assessments

At baseline and visit 3 (2005-2006), trained interviewers assessed the participants’ food and nutrient intake using validated semiquantitative food frequency questionnaires (FFQs) that listed 103 food items at the baseline visit and 106 food items at visit 3. All items were comparable between the 2 FFQs except for a few items (Supplementary Material 2). The validity and reproducibility of FFQs have been examined in detail elsewhere (reproducibility: 0.45 for median correlation coefficients between the first and second FFQ for nutrient intake; validity: 0.39 for de-attenuated correlation coefficients between the second FFQ and 12-day dietary records for nutrient intake) [25]. Participants were asked how frequently they consumed the average portion sizes of 103 or 106 food items during the past year with the help of food photographs for accuracy. There were 9 frequency categories (ranging from “never or rarely” to “3 times/day”) and three serving sizes (0.5, 1.0, and 1.5 standard portion size) on the FFQs to determine the frequency and the amount of consumption. Only at visit 3 were participants asked to indicate whether they ate each item for 3 months 9 months or 12 months of the year for food items with limited seasonal availability. Daily nutrient intakes were calculated using weighted frequencies per day and serving sizes per unit for each food item based on the nutrient database in the Seventh Edition of the Korean Food Composition Table [26].

In this study, we used 3 different approaches for analyzing repeated dietary measurements to compare the different effects of several temporal assumptions in diets on outcomes. For the most recent intake, we related the incidence of abdominal obesity between 2001-2002 and 2005-2006 to the dietary intake reported in 2001-2002 and the incidence of abdominal obesity between 2005-2006 and 2015-2016 to the dietary intake reported in 2005-2006 [27]. The cumulative average intake for each participant was calculated by averaging the dietary intakes at baseline and visit 3 up to the endpoint or censoring [27] (Supplementary Material 3). For each PDI, the baseline, most recent, and cumulative average scores were referred to as PDI_baseline_, PDI_recent_, and PDI_average_, respectively.

To assess the degree of adherence to plant‐based diets, we used 3 established plant‐based diet index scores (PDI, hPDI, and uPDI) using the given dietary data [20]; these indices were adapted from the original plant-based diet scale by Satija et al. [7]. We created 17 food groups based on nutrient and culinary similarities and classified them into 3 larger categories (healthy plant foods, less healthy plant foods, and animal foods) (Supplementary Material 4). From the original PDIs, 2 food groups were omitted because questions about “vegetable oil” were not asked at all and questions regarding “fruit juice” were not asked separately, and 1 new food group was added to consider that Koreans traditionally consume salted and pickled vegetables such as kimchi. Thus, healthy plant foods included whole grains, fruits, vegetables, nuts, legumes, and tea and coffee; less healthy plant foods included refined grains, potatoes, sugar-sweetened beverages, sweets and desserts, and salted vegetables; and animal foods included animal fat, dairy, eggs, fish or seafood, meat, and miscellaneous animal foods. After adjusting for total energy intake using the residual method [23], the 17 food groups were divided into quintiles of consumption and received a score between 1 and 5. For positive scores, we gave a score of 5 to participants in the highest consumption quintile of a food group and a score of 1 to those in the lowest consumption quintile of a food group. For reverse scores, we gave a score of 1 to participants in the highest quintile of a food group and a score of 5 to those in the lowest quintile of a food group. The overall PDI was created by assigning positive scores to all plant food groups and reverse scores to the animal food groups. The hPDI was created by giving positive scores to healthy plant food groups and reverse scores to less healthy plant food groups and animal food groups. The uPDI was created by assigning positive scores to less healthy plant food groups and reverse scores to healthy plant food groups and animal food groups. Finally, we summed each food group score (possible ranges in this study: 30-73 for the PDI, 30-71 for the hPDI, and 29-75 for the uPDI).

### Measurement of waist circumference and

ascertainment of incident abdominal obesity WC was measured biennially by trained measurers until 2016. Before the examination at each visit, the same coordinator trained the measurers using videos and hands-on training based on standardized protocols for examinations. WC was measured at the halfway point between the lowest rib margin and the iliac crest. Abdominal obesity was defined based on the WC measurement. Among participants with a WC less than 90 cm for men and 85 cm for women at the baseline survey, we defined incident cases of abdominal obesity as individuals with a WC above the determined cut-off level of the Korean Society for the Study of Obesity (≥ 90 cm for men and ≥ 85 cm for women) in follow-up examinations [28].

### Assessment of covariates

The trained interviewers and measurers collected data based on standardized protocols for questionnaire surveys and examinations.

#### General characteristics based on questionnaires

The structured questionnaires included comprehensive information on demographics (age, gender, education level) and lifestyle (physical activity level, smoking status, drinking status, and alcohol consumption). A high school education indicated 12 years of schooling or more, and the physical activity level was presented as metabolic equivalent of task (MET) per day. For smoking status, participants were asked if they had ever smoked, with 3 response options (1= never, 2= former, 3= current). For drinking status, participants were asked if they currently drank alcohol, with 3 response options (1 = never, 2 = former, 3 = current). Participants were categorized according to smoking and drinking status as current smokers or non-smokers (including former smokers) and current drinkers or non-drinkers (including former drinkers), respectively. To estimate daily alcohol consumption, current drinkers were further asked about the average frequency and average number of servings of alcoholic beverages that are commonly consumed in Korea (soju, takju, beer, refined rice wine, wine, and whisky) in the preceding year, and total daily alcohol consumption was calculated based on the total volume of all alcoholic beverages consumed, as expressed in grams of alcohol per day (g/day).

#### Anthropometric measurements

Height was measured with a standard height scale to the nearest 0.1 cm, and weight was measured with a metric weight scale to the nearest 0.01 kg with the participants in light clothing without shoes. We calculated BMI using the ratio of weight (kg) to height squared (m^2^).

### Statistical analysis

All PDI scores were divided into quintiles for further analyses. We presented age-adjusted and gender-adjusted estimates of the participants’ baseline characteristics according to each PDI score quintile using a general linear model. To examine the associations between the quintiles of each PDI score and abdominal obesity incidence, we applied Cox proportional-hazard regression and presented them as hazard ratios (HRs) and 95% confidence intervals (CIs). The person-time of follow-up was calculated from the date of the baseline examination until the date of incident abdominal obesity, censoring, or the date of the last examination, whichever came first. The median follow-up time was 9.0 years. A multivariable model was adjusted for age (years), gender (men or women), total energy intake (kcal/day), high school graduate (yes or, no), physical activity level (METs), current smoking (yes or no), alcohol intake (g/day), and BMI. To test for potential linear trends, we treated each median PDI score in each quintile as a continuous variable. Since possible differences in dietary habits between men and women may exist, we tested for the interaction effect of gender by including cross-product terms of each PDI score and gender (men or women).

As a sensitivity analysis, we repeated the main analyses (1) after classifying salted vegetables into the healthy plant food category; (2) after excluding incident cases of abdominal obesity occurring within the first 2 follow-up years (516 participants were excluded, n= 5,538); and (3) after excluding incident cases of hypertension, type 2 diabetes, dyslipidemia, and general obesity that occurred before developing abdominal obesity (398 participants were excluded, n= 5,656). All data were analyzed with SAS version 9.4 (SAS Institute Inc., Cary, NC, USA), and an α level of 0.05 was considered statistically significant.

### Ethics statement

This study protocol was approved by the Insititutional Review Board of Hallym University (HIRB-2021-087).

## RESULTS

As shown in Table 1, those in the highest quintiles of PDI_average_ and HR_average_ tended to be older, less educated, non-smokers, and more physically active, and consume a higher total energy intake. In contrast, those in the highest quintile of uPDI_average_ tended to be older, less educated, current smokers, and more physically active, have a slightly lower BMI, and consume a higher total energy intake. When using the baseline and most recent diet measures, similar trends in these characteristics were observed (Supplementary Materials 5 and 6).

We confirmed incident abdominal obesity in 880 men and 898 women during a follow-up of up to 12.6 years (45,818 person-years). For PDI and hPDI, there were no meaningful associations across all approaches. All uPDIs were positively associated with abdominal obesity risk in all approaches (all p-values for trends < 0.001), and slightly stronger estimates were observed when using the cumulative average diet than either the baseline or most recent diet (Q5 vs. Q1, uPDI_baseline_: HR, 1.70; 95% CI, 1.46 to 1.98; uPDI_recent_: HR, 1.52; 95% CI, 1.30 to 1.78; uPDI_average_: HR, 1.76; 95% CI, 1.51 to 2.06) (Table 2 and Supplementary Material 7).

In the gender-stratified analyses, PDI showed no meaningful associations with abdominal obesity risk across all 3 approaches in men or women. In all approaches, uPDI was positively associated with abdominal obesity risk (all p-values for trends < 0.05), and slightly stronger estimates were observed when using the cumulative average diet than either the baseline or most recent diet in both men and women. In men, only HR_average_ was inversely associated with abdominal obesity risk (HR_Q5_ vs. Q1, 0.77; 95% CI, 0.62 to 0.96; p for trend= 0.040). In women, hPDI_baseline_ and HR_average_ were positively associated with abdominal obesity risk (Q5 vs. Q1, hPDI_baseline_: HR, 1.30; 95% CI, 1.04 to 1.62, p for trend= 0.004; HR_average_: HR, 1.13; 95% CI, 0.91 to 1.40, p for trend= 0.040) (Table 3).

In all sensitivity analyses, the associations remained similar to the main findings when salted vegetables were classified as belonging to the healthy plant food category (Supplementary Material 8), incident cases of abdominal obesity during the first 2 follow-up years were excluded (Supplementary Material 9), and incident cases of other chronic diseases were excluded (Supplementary Material 10).

## DISCUSSION

In this prospective study of 6,054 Korean participants, we observed linear positive associations between uPDI and abdominal obesity risk irrespective of demographic and lifestyle characteristics. Although the strength of the findings varied slightly, the substantive results were consistent across all approaches (baseline diet only, most recent diet, or cumulative average diet) in both total and gender-stratified analyses. PDI showed no meaningful association with abdominal obesity risk in all analyses. HR_average_ had an inverse association with abdominal obesity risk in men, whereas hPDI_baseline_ had a positive association with abdominal obesity risk in women.

In this study, we observed that greater adherence to a diet rich in refined grains, sugars, and salted vegetables, represented by the uPDI, was linearly associated with a 16-24% higher risk of abdominal obesity. These results are in line with previous studies that specifically focused on abdominal adiposity. In prospective studies, greater adherence to plant-based diets decreased WC by 2.0 cm over 7 years [29], and individuals with higher uPDI scores had a 1.46 times greater risk of abdominal obesity when using WC as a component of metabolic syndrome [20]. In cross-sectional studies, individuals with higher hPDI scores had lower WCs (92.9, 86.8, and 87.6 cm in each tertile) [19] and greater reductions in visceral abdominal adipose tissue volume (-4.9% per 10-point increase in hPDI score) [17], and individuals with higher uPDI scores had 1.54 times to 2.36 times greater odds of abdominal obesity [21,22].

To our knowledge, this is the first study to examine the association of adherence to plant-based diets with abdominal obesity incidence using 3 different exposure time points in Korea. The use of repeated diet measures allows us to reduce measurement error and to examine the different hypothesized temporal assumptions between exposures and outcomes [23]. As analytic strategies, we used baseline, most recent, and cumulative average dietary measures [23]. When using the baseline diet, we hypothesized a long induction effect of diet, which assumes an unchanged or constant rate of change in diet during follow-up [23]. When using the most recent diet, we hypothesized a relatively short induction effect compared to the use of the baseline diet [23]. When using the cumulative average diet, we hypothesized the cumulative effect of a longterm diet by considering possible changes in participants’ diet during follow-up [23]. Although the strength of the findings was slightly stronger when using the cumulative average measurements, the positive associations between uPDI scores and abdominal obesity incidence were consistent across all 3 approaches. These findings may suggest that the management of unhealthy plant food consumption is helpful for preventing abdominal obesity irrespective of the length of the induction period. Further studies with repeated dietary measurements with longer follow-up periods are required for further improvement.

A possible explanation for positive associations of all uPDIs in relation to abdominal obesity risk includes the different nutritional characteristics according to the levels of uPDI. As opposed to the PDI and hPDI quintiles, participants in the fifth quintile of uPDI in all 3 measures consumed less iron, potassium, vitamin C, folate, beta-carotene, and fiber than those in the first quintile. The possible roles of these nutrients in the mechanisms of adiposity are as follows: (1) lower iron intake may deregulate the iron balance in white adipose tissue and inhibit adaptive thermogenesis [30]; (2) dietary vitamin C may inhibit visceral adipocyte hypertrophy and glucose intolerance by suppressing the gene expression involved in lipogenesis [31]; (3) dietary folate may inhibit visceral adipose tissue accumulation by reducing oxidative stress [32] or genetic regulation at the level of adipose tissue [33]; (4) beta-carotene may contribute to obesity prevention by reducing oxidative stress [34]; and (5) dietary fiber may reduce visceral fat mass by promoting adipocyte lipolysis in white adipose tissue and enhancing white adipose tissue browning via the activation of protein expression involved in thermogenesis [35]. Our findings may reflect the consequences of mixed effects of each single component. Besides these nutrients, some food components (e.g., phytochemicals, polyphenols, or probiotics) may contribute to the biological mechanism underlying the relationship between uPDI and obesity [36-38]. Since the relevant data for these components were not available in our data, future research is required to address this issue.

In the present study, HR_average_ was associated with a lower risk of abdominal obesity in men, whereas hPDI_baseline_ was associated with a greater risk of abdominal obesity in women. This discrepancy may be partially explained by the fact that women consume more total sugar than men due to a higher fruit consumption. In our sample, the mean fruit consumption was 1.4 servings/day (range: 0.0-12.6) for men and 1.8 servings/day (range: 0.0-24.4) for women. In addition, the proportion of those whose total sugar intake was greater than 20% of the total energy intake (recommended level: 10-20% of total energy intake) was 9% for men and 16% for women. Alternatively, it is possible that confounding from unknown factors, such as gender differences in the gut microbiome composition [39], may hinder the detection of such associations. Further studies are warranted to resolve this issue.

The present study has the following strengths. First, our findings were based on well-designed large-scale prospective cohort data with a study population of approximately 6,000 individuals and a long-term follow-up of up to 12 years. The long-term follow-up in this study allowed us to identify enough new cases of abdominal obesity, which takes a relatively long time to develop. Second, we examined both the beneficial and harmful effects of salted vegetables such as kimchi by classifying them into the less healthy category (main results) or the healthy category (alternative results, Supplementary Material 8). Based on the similar findings in both analyses, we can assume a neutral effect of salted vegetables in our study population, and our findings may be comparable to those of other populations of individuals who do not consume salted vegetables on a daily basis.

Strengths aside, several limitations should be considered when interpreting the results of this study. First, our findings may not be applicable to other study settings or populations with different ages, races/ethnicities, and locations. Specifically, compared to study participants, those who enrolled in the KoGES study but were excluded from the final analytic set were more often women, older participants, and rural area residents, and were less likely to have higher education and higher income levels. Thus, the results should be interpreted with caution. Second, the misclassification of abdominal obesity may have occurred because WC may not fully capture visceral abdominal adiposity compared to medical imaging techniques. However, WC has been considered a validated and simple marker of abdominal obesity [8,9], and we observed a strong correlation between WC and body fat percentage using bioelectrical impedance analysis in our cohort (correlation coefficient: 0.8 in men; 0.7 in women). Third, FFQs are generally prone to recall bias and inaccurate portion size measurements [23]. Furthermore, 2 dietary measurements were available, which may not have been sufficient to definitively present different induction periods. For example, if abdominal obesity was identified in the last visit (2015-2016), then it is uncertain whether the participant’s diet was stable throughout the follow-up period after the third visit (2005-2006). In addition, fruit juices could not be assessed because fruits and fruit juices were assessed using a single aggregated item; furthermore, in our FFQs, barley or mixed grains were usually consumed with white rice, and the classification of whole grains may not have been accurate. Fourth, possible uncontrolled confounding factors may exist, and unknown factors related to abdominal adiposity may have affected the direction and/or size of the association. Fifth, potential reverse causation may have biased the results due to possible changes in the dietary habits of the study participants. However, this concern is relatively unlikely based on the similar results in our sensitivity analyses (Supplementary Materials 9 and 10).

In conclusion, greater adherence to unhealthy plant-based diets may increase the risk of incident abdominal obesity among Korean adults aged 40-69 years. Although 3 different approaches for analyzing repeated dietary measurements were examined, the substantive findings were generally consistent across all approaches (i.e., using the baseline, most recent, or cumulative average diets). Future larger-scale observational studies with more repeated dietary measurements are warranted to determine whether the results are replicable and to provide more evidence for recommendations.

## Figures and Tables

**Table 1.** Age- and gender-adjusted baseline characteristics of the study participants according to 3 different plant-based diet indices (n=6,054)

Characteristics		Plant-based diet index			p for trend^1^	Healthy plant-based diet index			p for trend^1^	Unhealthy plant-based diet index			p for trend^1^
		Q1	Q3	Q5		Q1	Q3	Q5		Q1	Q3	Q5	
Baseline diet only													
	Total (n)	1,181	1,357	1,182		1,242	1,082	1,250		1,270	1,091	1,267	
	Median of each exposure (Min, Max)	44.0 (30.0, 46.0)	51.0 (50.0, 52.0)	58.0 (56.0, 73.0)		43.0 (30.0, 45.0)	51.0 (50.0, 52.0)	59.0 (57.0, 71.0)		43.0 (29.0, 45.0)	51.0 (50.0, 52.0)	60.0 (57.0, 74.0)	
	Age (yr)	49.5±0.3	51.1±0.2	52.4±0.3	<0.001	48.6±0.2	50.8±0.3	53.5±0.2	<0.001	49.4±0.2	50.5±0.3	53.1±0.2	<0.001
	Elderly, ≥65 yr	7.6	11.2	14.2	0.001	6.2	10.5	16.1	<0.001	7.2	9.8	16.4	<0.001
	Men	54.5	54.3	55.1	0.790	54.5	53.3	54.6	0.520	52.8	54.2	53.4	0.300
	Area, Ansan	64.8	63.5	52.8	<0.001	74.9	60.3	49.5	<0.001	74.1	64.6	41.2	<0.001
	High school graduate	54.9	50.9	42.7	<0.001	51.9	50.0	45.7	<0.001	59.9	49.2	38.5	<0.001
	Current smoker	27.1	27.2	28.9	0.330	27.5	26.9	25.8	0.110	24.5	27.3	28.9	0.001
	Alcohol intake (g/day)	10.0±0.6	9.6±0.6	10.7±0.6	0.980	9.2±0.6	9.2±0.6	11.1±0.6	0.070	10.7±0.6	9.4±0.6	9.9±0.6	0.400
	Physical activity level (MET/day)	21.3±0.4	22.8±0.4	24.3±0.4	<0.001	20.9±0.4	23.3±0.4	24.7±0.4	<0.001	20.7±0.4	22.1±0.4	25.8±0.4	<0.001
	BMI (kg/m^2^)	23.4±0.1	23.5±0.1	23.5±0.1	0.070	23.5±0.1	23.4±0.1	23.5±0.1	0.500	23.6±0.1	23.5±0.1	23.3±0.1	<0.001
	WC (cm)	78.1±0.2	78.2±0.2	78.4±0.2	0.020	77.9±0.2	78.1±0.2	78.8±0.2	<0.001	78.1±0.2	78.4±0.2	78.4±0.2	0.230
	Healthy plant foods (servings/day)^2^	7.92±0.13	9.55±0.12	11.44±0.13	<0.001	7.53±0.13	9.55±0.14	11.88±0.13	<0.001	13.40±0.11	9.61±0.12	6.16±0.11	<0.001
	Less healthy plant foods (servings/day)^3^	6.81±0.09	7.96±0.08	9.51±0.09	<0.001	10.12±0.08	7.91±0.09	5.94±0.08	<0.001	6.76±0.09	7.98±0.09	9.37±0.09	<0.001
	Animal foods (servings/day)^4^	4.29±0.06	3.52±0.06	2.76±0.06	<0.001	4.78±0.06	3.64±0.06	2.20±0.06	<0.001	5.19±0.05	3.45±0.06	2.06±0.05	<0.001
	Energy intake (kcal/day)	1,782±15	1,892 ±14	1,979±15	<0.001	1,714±14	1,877±15	2,077±14	<0.001	1,701±14	1,910±15	2,072±14	<0.001
Most recent diet													
	Total (n)	1,138	1,358	1,134		1,182	1,154	1,161		1,275	1,033	1,198	
	Median of each exposure (Min, Max)	44.0 (30.0, 46.0)	51.0 (50.0, 52.0)	58.0 (56.0, 73.0)		43.0 (31.0, 45.0)	51.0 (50.0, 52.0)	59.0 (57.0, 71.0)		42.0 (29.0, 45.0)	51.0 (50.0, 52.0)	60.0 (57.0, 75.0)	
	Age (y)	50.0±0.3	50.8±0.2	51.9±0.3	<0.001	49.1±0.3	50.9±0.3	52.4±0.3	<0.001	49.1±0.2	51.1±0.3	52.6±0.2	<0.001
	Elderly, ≥65 yr	9.1	10.5	12.3	0.040	7.9	11.3	13.4	<0.001	6.7	12.1	13.9	<0.001
	Men	53.1	54.9	55.6	0.410	54.2	55.8	52.9	0.470	53.3	51.4	50.8	0.240
	Area, Ansan	61.3	63.0	58.6	0.410	65.5	58.7	56.6	<0.001	77.1	60.6	41.9	<0.001
	High school graduate	60.8	50.3	45.3	0.003	51.0	47.8	47.3	0.020	60.9	48.8	37.4	<0.001
	Current smoker	27.3	28.6	27.3	0.510	29.5	27.6	23.3	<0.001	25.4	28.7	28.9	0.001
	Alcohol intake (g/day)	10.3±0.6	9.9±0.6	8.8±0.6	0.020	10.4±0.6	10.1±0.6	9.6±0.6	0.550	10.5±0.6	10.4±0.7	9.6±0.6	0.440
	Physical activity level (MET/day)	21.7±0.4	22.2±0.4	24.0±0.4	<0.001	22.2±0.4	23.2±0.4	22.9±0.4	0.150	20.5±0.4	22.3±0.4	26.1±0.4	<0.001
	BMI (kg/m^2^)	23.4±0.1	23.4±0.1	23.5±0.1	0.120	23.4±0.1	23.4±0.1	23.6±0.1	0.010	23.6±0.1	23.4±0.1	23.2±0.1	<0.001
	WC (cm)	78.4±0.2	78.2±0.2	78.2±0.2	0.670	78.0±0.2	78.1±0.2	78.7±0.2	<0.001	78.1±0.2	78.3±0.2	78.4±0.2	0.090
	Healthy plant foods (servings/day)^2^	7.89±0.13	9.86±0.12	12.17±0.13	<0.001	8.32±0.13	9.86±0.13	11.60±0.13	<0.001	13.73±0.11	9.84±0.11	6.26±0.11	<0.001
	Less healthy plant foods (servings/day)^3^	5.90±0.10	7.23±0.09	8.77±0.10	<0.001	9.63±0.09	7.06±0.09	5.28±0.09	<0.001	6.57±0.10	7.24±0.11	8.09±0.10	<0.001
	Animal foods (servings/day)^4^	3.84±0.08	3.50±0.07	2.91±0.08	<0.001	5.06±0.07	3.41±0.07	2.07±0.07	<0.001	5.37±0.07	3.30±0.07	1.84±0.07	<0.001
	Energy intake (kcal/day)	1,751±16	1,840±14	1,861±16	<0.001	1,640±15	1,826±15	1,966±15	<0.001	1,586±14	1,835±16	2,051±15	<0.001
Cumulative average													
	Total (n)	1,260	1,431	1,263		1,236	1,169	1,224		1,228	1,176	1,226	
	Median of each exposure (Min, Max)	45.5 (30.0, 47.0)	51.0 (50.0, 52.0)	57.0 (55.0, 73.0)		44.0 (31.0, 46.0)	51.0 (50.0, 52.0)	58.0 (56.0, 70.0)		43.5 (30.0, 46.0)	51.0 (49.5, 52.0)	59.0 (56.0, 71.0)	
	Age (yr)	50.6±0.2	50.6±0.2	52.2±0.2	<0.001	48.6±0.2	50.9±0.3	53.2±0.2	<0.001	49.2±0.2	50.5±0.2	53.3±0.2	<0.001
	Elderly, ≥65 yr	9.1	9.1	12.7	0.001	6.7	10.5	15.0	<0.001	6.7	10.0	16.8	<0.001
	Men	55.4	55.4	53.6	0.860	54.3	52.3	53.7	0.510	56.7	55.8	56.8	0.610
	Area, Ansan	63.2	62.2	55.1	<0.001	72.0	60.0	53.4	<0.001	78.0	63.4	37.7	<0.001
	High school graduate	54.2	50.2	43.6	<0.001	52.3	48.0	47.6	0.002	61.4	49.0	36.7	<0.001
	Current smoker	28.4	27.7	29.5	0.180	29.4	26.0	24.6	0.001	25.1	27.7	30.1	<0.001
	Alcohol intake (g/day)	11.0±0.6	9.2±0.6	9.8±0.6	0.050	10.1±0.6	9.7±0.6	10.5±0.6	0.430	10.4±0.6	10.1±0.6	9.8±0.6	0.550
	Physical activity level (MET/day)	21.4±0.4	22.8±0.4	24.5±0.4	<0.001	21.5±0.4	23.1±0.4	23.6±0.4	0.020	20.0±0.4	22.6±0.4	26.2±0.4	<0.001
	BMI (kg/m^2^)	23.5±0.1	23.4±0.1	23.6±0.1	0.060	23.4±0.1	23.3±0.1	23.6±0.1	0.080	23.6±0.1	23.5±0.1	23.3±0.1	<0.001
	WC (cm)	78.4±0.2	78.2±0.2	78.5±0.2	0.300	77.9±0.2	77.9±0.2	78.9±0.2	<0.001	78.2±0.2	78.1±0.2	78.6±0.2	0.050
	Healthy plant foods (servings/day)^2^	8.12±0.11	9.71±0.10	11.54±0.11	<0.001	8.12±0.11	9.62±0.11	11.65±0.11	<0.001	13.02±0.10	9.82±0.10	6.57±0.10	<0.001
	Less healthy plant foods (servings/day)^3^	6.67±0.08	7.59±0.07	8.98±0.08	<0.001	9.59±0.08	7.57±0.08	5.87±0.08	<0.001	6.72±0.08	7.53±0.08	8.76±0.08	<0.001
	Animal foods (servings/day)^4^	4.04±0.06	3.54±0.05	2.91±0.06	<0.001	4.83±0.06	3.52±0.06	2.34±0.06	<0.001	5.03±0.05	3.49±0.05	2.07±0.05	<0.001
	Energy intake (kcal/day)	1,777±13	1,864±12	1,921±13	<0.001	1,724±13	1,852±13	2,003±13	<0.001	1,690±13	1,858±13	2,013±13	<0.001

Values are presented as mean±standard error or %. Q, quintile; KRW, Korean won; MET, metabolic equivalent of task; BMI, body mass index; WC, waist circumference; Min, minimum; Max, maximum.

1 Treating the median value of each group as a continuous variable using a general linear model.

2 Healthy plant foods included whole grains, fruits, vegetables, nuts, legumes, tea, and coffee.

3 Less healthy plant foods included refined grains, potatoes, sugar-sweetened beverages, sweets and desserts, and salty foods.

4 Animal foods included animal fat, dairy, eggs, fish, meat, and miscellaneous animal foods.

**Table 2.** Associations between plant-based diet indices and abdominal obesity incidence (n=6,054)

Variables		Baseline diet only		Most recent diet		Cumulative average	
		No. of cases/person-yr	Multivariable-adjusted^1^	No. of cases/person-yr	Multivariable-adjusted^1^	No. of cases/person-yr	Multivariable-adjusted^1^
PDI							
	Q1	321/9,013	1.00 (reference)	343/8,503	1.00 (reference)	379/8,812	1.00 (reference)
	Q2	351/9,025	1.13 (0.97, 1.31)	352/9,392	1.00 (0.86, 1.16)	298/9,013	0.74 (0.64, 0.87)
	Q3	372/10,406	0.93 (0.80, 1.08)	395/10,307	0.94 (0.82, 1.09)	403/11,066	0.79 (0.68, 0.90)
	Q4	353/8,603	0.97 (0.83, 1.13)	342/9,199	0.85 (0.73, 0.99)	272/8,259	0.66 (0.56, 0.77)
	Q5	381/8,771	1.06 (0.92, 1.24)	346/8,418	0.94 (0.81, 1.10)	426/8,667	0.94 (0.82, 1.08)
	p for trend^2^		0.990		0.119		0.379
hPDI							
	Q1	318/9,778	1.00 (reference)	339/8,903	1.00 (reference)	343/8,874	1.00 (reference)
	Q2	340/9,406	1.11 (0.95, 1.29)	344/9,785	0.86 (0.74, 1.00)	328/9,793	0.77 (0.66, 0.90)
	Q3	322/8,256	1.09 (0.93, 1.27)	333/9,006	0.82 (0.71, 0.96)	341/9,220	0.83 (0.72, 0.97)
	Q4	400/9,218	1.24 (1.06, 1.44)	394/9,752	0.87 (0.75, 1.01)	371/9,567	0.86 (0.74, 1.00)
	Q5	398/9,160	1.09 (0.94, 1.27)	368/8,372	0.97 (0.83, 1.12)	395/8,363	0.94 (0.81, 1.09)
	p for trend^2^		0.110		0.826		0.963
uPDI							
	Q1	304/10,259	1.00 (reference)	321/10,345	1.00 (reference)	306/9,633	1.00 (reference)
	Q2	336/9,661	1.17 (1.00, 1.37)	335/9,979	1.06 (0.91, 1.23)	327/9,616	1.06 (0.90, 1.24)
	Q3	321/8,280	1.34 (1.14, 1.57)	303/7,834	1.20 (1.02, 1.41)	334/9,237	1.11 (0.95, 1.30)
	Q4	364/8,833	1.34 (1.14, 1.56)	418/9,291	1.37 (1.18, 1.59)	357/9,320	1.15 (0.98, 1.35)
	Q5	453/8,786	1.70 (1.46, 1.98)	401/8,369	1.52 (1.30, 1.78)	454/8,012	1.76 (1.51, 2.06)
	p for trend^2^		<0.001		<0.001		<0.001

Values are presented as hazard ratio (95% confidence interval). Q, quintile; PDI, plant-based diet index; hPDI, healthy plant-based diet index; uPDI, unhealthy plant-based diet index; MET, metabolic equivalent of task.

1 The multivariable-adjusted model was adjusted for age (years), gender (men or women), total energy intake (kcal/day), high school graduate (yes or no), physical activity level (METs), current smoking (yes or no), alcohol intake (g/day), and body mass index at baseline.

2 Treating the median value of each group as a continuous variable using a Cox proportional-hazard model.

**Table 3.** Associations between plant-based diet indices and abdominal obesity incidence: stratified by gender (n=6,054)

Multivariable-adjusted model^1^		Baseline diet only		Most recent diet		Cumulative average	
		Men (n=3,273)	Women (n=2,781)	Men (n=3,273)	Women (n=2,781)	Men (n=3,273)	Women (n=2,781)
PDI							
	Q1	1.00 (reference)	1.00 (reference)	1.00 (reference)	1.00 (reference)	1.00 (reference)	1.00 (reference)
	Q2	1.15 (0.93, 1.42)	1.09 (0.87, 1.35)	1.08 (0.87, 1.33)	0.92 (0.74, 1.14)	0.76 (0.61, 0.95)	0.73 (0.58, 0.90)
	Q3	0.88 (0.71, 1.09)	0.98 (0.79, 1.20)	0.92 (0.75, 1.13)	0.97 (0.79, 1.19)	0.76 (0.62, 0.92)	0.84 (0.69, 1.03)
	Q4	0.99 (0.80, 1.23)	0.94 (0.75, 1.16)	0.88 (0.71, 1.09)	0.83 (0.68, 1.03)	0.61 (0.48, 0.76)	0.70 (0.56, 0.87)
	Q5	1.00 (0.80, 1.24)	1.14 (0.92, 1.41)	0.94 (0.76, 1.17)	0.95 (0.77, 1.18)	0.93 (0.76, 1.13)	0.97 (0.80, 1.18)
	p for trend^2^	0.610	0.530	0.210	0.430	0.320	0.970
	p for interaction^3^	0.640		0.870		0.640	
hPDI							
	Q1	1.00 (reference)	1.00 (reference)	1.00 (reference)	1.00 (reference)	1.00 (reference)	1.00 (reference)
	Q2	1.05 (0.85, 1.29)	1.16 (0.92, 1.46)	0.76 (0.62, 0.94)	0.89 (0.72, 1.12)	0.72 (0.58, 0.88)	0.80 (0.64, 1.01)
	Q3	0.88 (0.70, 1.09)	1.38 (1.11, 1.73)	0.63 (0.51, 0.78)	1.08 (0.87, 1.34)	0.66 (0.53, 0.81)	0.998 (0.81, 1.24)
	Q4	1.03 (0.83, 1.28)	1.48 (1.20, 1.83)	0.69 (0.56, 0.85)	1.06 (0.86, 1.31)	0.71 (0.58, 0.88)	1.04 (0.84, 1.29)
	Q5	0.93 (0.75, 1.16)	1.30 (1.04. 1.62)	0.82 (0.67, 1.02)	1.09 (0.88, 1.36)	0.77 (0.62, 0.96)	1.13 (0.91, 1.40)
	p for trend^2^	0.520	0.004	0.050	0.180	0.040	0.040
	p for interaction^3^	0.001		0.002		<0.001	
uPDI							
	Q1	1.00 (reference)	1.00 (reference)	1.00 (reference)	1.00 (reference)	1.00 (reference)	1.00 (reference)
	Q2	1.14 (0.92, 1.41)	1.18 (0.94, 1.48)	0.99 (0.80, 1.22)	1.17 (0.93, 1.47)	1.18 (0.95, 1.46)	1.02 (0.81, 1.28)
	Q3	1.24 (0.99, 1.55)	1.42 (1.13, 1.79)	1.07 (0.85, 1.34)	1.37 (1.08, 1.73)	1.07 (0.86, 1.32)	1.20 (0.95, 1.52)
	Q4	1.16 (0.93, 1.44)	1.50 (1.20, 1.88)	1.26 (1.03, 1.54)	1.47 (1.17, 1.85)	0.95 (0.76, 1.19)	1.31 (1.04, 1.65)
	Q5	1.51 (1.22, 1.87)	1.81 (1.45, 2.26)	1.22 (0.97, 1.52)	1.80 (1.43, 2.26)	1.60 (1.30, 1.98)	1.90 (1.50, 2.39)
	p for trend^2^	<0.001	<0.001	0.010	<0.001	0.001	<0.001
	p for interaction^3^	0.004		<0.001		<0.001	

Values are presented as hazard ratio (95% confidence interval). Q, quintile; PDI, plant-based diet index; hPDI, healthy plant-based diet index; uPDI, unhealthy plant-based diet index; MET, metabolic equivalent of task.

1 The multivariable-adjusted model was adjusted for age (years), gender (men or women), total energy intake (kcal/day), high school graduate (yes or no), physical activity level (METs), current smoking (yes or no), alcohol intake (g/day), and body mass index at baseline.

2 Treating the median value of each group as a continuous variable using a Cox proportional-hazard model.

3 Including the cross-product term of PDIs and gender in the multivariable model.

## SUPPLEMENTARY MATERIALS

Supplementary Material 1. Participant flow chart

Supplementary Material 2 Food items listed in the FFQ with 103 items and the FFQ with 106 items in the KoGES_Ansan Ansung Study

Supplementary Material 3 Illustration of the three approaches for analyzing repeated dietary measurements in the KoGES Ansan and Ansung Study

Supplementary Material 4 Food items constituting the 17 food groups using the KoGES_Ansan Ansung Study

Supplementary Material 5 Age- and sex-adjusted nutritional characteristics of the study participants according to 3 different plant-based diet indices (n=6054)

Supplementary Material 6 Age- and sex-adjusted food group intakes of the study participants according to 3 different plant-based diet indices (n=6054)

Supplementary Material 7 Adjusted HRs and 95% CIs for incident abdominal obesity according to the continuous uPDIs using restricted cubic splines

Supplementary Material 8 Hazard ratios (HRs) and 95% confidence intervals (CIs) for abdominal obesity according to the plant-based diet indices with salted vegetables categorized into the healthy plant food group (n=6054)

Supplementary Material 9 Hazard ratios (HRs) and 95% confidence intervals (CIs) for abdominal obesity according to plant-based diet indices after excluding incident cases of abdominal obesity occurring within the first two follow-up years (n=5538)

Supplementary Material 10 Hazard ratios (HRs) and 95% confidence intervals (CIs) for abdominal obesity according to the plant-based diet indices after excluding incident cases of hypertension, T2DM, dyslipidemia, and general obesity occurring before the development of abdominal obesity during the follow-up (n=5656)
