# Supplementary material for: Positive association of unhealthy plant-based diets with the incidence of abdominal obesity in Korea: a comparison of baseline, most recent, and cumulative average diets
Source: Epidemiol Health. 2022 Aug 2;44:e2022063. doi: 10.4178/epih.e2022063 (PMC9754918; doi:10.4178/epih.e2022063)
Supplement: Supplementary Material 1. — Participant flow chart [file epih-44-e2022063-suppl1.docx]

**Online supplementary material**

This appendix is a part of the original submission and has been peer reviewed.

Supplement to: Jung S and Park S. Positive association of unhealthy plant-based diets with the incidence of abdominal obesity: a comparison of baseline, most recent, and cumulative average diets

**Supplementary Material 1. Participant flow chart**

**Supplementary Material 2. Food items listed in the FFQ with 103 items and the FFQ with 106 items in the KoGES_Ansan Ansung Study**

**Supplementary Material 3. Illustration of the three approaches for analyzing repeated dietary measurements in the KoGES Ansan and Ansung Study**

**Supplementary Material 4. Food items constituting the 17 food groups using the KoGES_Ansan Ansung Study**

**Supplementary Material 5. Age- and sex-adjusted nutritional characteristics of the study participants according to 3 different plant-based diet indices (n=6054)**

**Supplementary Material 6. Age- and sex-adjusted food group intakes of the study participants according to 3 different plant-based diet indices (n=6054)**

**Supplementary Material 7. Adjusted HRs and 95% CIs for incident abdominal obesity according to the continuous uPDIs using restricted cubic splines**

**Supplementary Material 8. Hazard ratios (HRs) and 95% confidence intervals (CIs) for abdominal obesity according to the plant-based diet indices with salted vegetables categorized into the healthy plant food group (n=6054)**

**Supplementary Material 9. Hazard ratios (HRs) and 95% confidence intervals (CIs) for abdominal obesity according to plant-based diet indices after excluding incident cases of abdominal obesity occurring within the first two follow-up years (n=5538)**

**Supplementary Material 10. Hazard ratios (HRs) and 95% confidence intervals (CIs) for abdominal obesity according to the plant-based diet indices after excluding incident cases of hypertension, T2DM, dyslipidemia, and general obesity occurring before the development of abdominal obesity during the follow-up (n=5656)**

Baseline cycle of the KoGES Ansan & Ansung study (2001-2002)

(n=10,030)

Baseline waist circumference ≥90 cm for men and ≥85 cm for women

(n=2,974)

Baseline waist circumference <90 cm for men and <85 cm for women

(n=7,056)

Total energy intake at baseline <800 or >4000 kcal/d for men and <500 or >3500 kcal/d for women or incomplete dietary intake

(n=387)

Valid and complete dietary intake at baseline

(n=6,669)

Histories of heart diseases, stroke and/or cancer at baseline (n=318)

(n=17,135)

No histories of severe diseases

(n=6,351)

Missing information on key variables

(n=297)

(n=17,135)

**Final analytic sample**

**(n=6,054)**

**Supplementary Material 1. Participant flow chart.**
