# Supplementary material for: Positive association of unhealthy plant-based diets with the incidence of abdominal obesity in Korea: a comparison of baseline, most recent, and cumulative average diets
Source: Epidemiol Health. 2022 Aug 2;44:e2022063. doi: 10.4178/epih.e2022063 (PMC9754918; doi:10.4178/epih.e2022063)
Supplement: Supplementary Material 2 — Food items listed in the FFQ with 103 items and the FFQ with 106 items in the KoGES_Ansan Ansung Study [file epih-44-e2022063-suppl2.docx]

**Online supplementary material**

This appendix is a part of the original submission and has been peer reviewed.

Supplement to: Jung S and Park S. Positive association of unhealthy plant-based diets with the incidence of abdominal obesity: a comparison of baseline, most recent, and cumulative average diets

**Supplementary Material 1. Participant flow chart**

**Supplementary Material 2. Food items listed in the FFQ with 103 items and the FFQ with 106 items in the KoGES_Ansan Ansung Study**

**Supplementary Material 3. Illustration of the three approaches for analyzing repeated dietary measurements in the KoGES Ansan and Ansung Study**

**Supplementary Material 4. Food items constituting the 17 food groups using the KoGES_Ansan Ansung Study**

**Supplementary Material 5. Age- and sex-adjusted nutritional characteristics of the study participants according to 3 different plant-based diet indices (n=6054)**

**Supplementary Material 6. Age- and sex-adjusted food group intakes of the study participants according to 3 different plant-based diet indices (n=6054)**

**Supplementary Material 7. Adjusted HRs and 95% CIs for incident abdominal obesity according to the continuous uPDIs using restricted cubic splines**

**Supplementary Material 8. Hazard ratios (HRs) and 95% confidence intervals (CIs) for abdominal obesity according to the plant-based diet indices with salted vegetables categorized into the healthy plant food group (n=6054)**

**Supplementary Material 9. Hazard ratios (HRs) and 95% confidence intervals (CIs) for abdominal obesity according to plant-based diet indices after excluding incident cases of abdominal obesity occurring within the first two follow-up years (n=5538)**

**Supplementary Material 10. Hazard ratios (HRs) and 95% confidence intervals (CIs) for abdominal obesity according to the plant-based diet indices after excluding incident cases of hypertension, T2DM, dyslipidemia, and general obesity occurring before the development of abdominal obesity during the follow-up (n=5656)**

**Supplementary Material 2. Food items listed in the FFQ with 103 items and the FFQ with 106 items in the KoGES_Ansan Ansung Study**

|  | Item number |  | Difference |
| --- | --- | --- | --- |
| Food label | Baseline | Visit 3 |  |
| White rice | F001 | F001 | N |
| Barley rice | F002 | - | Included as mixed grain rice in visit 3 |
| Mixed grain rice | F003 | F003/F005 | N |
| Roasted grains powder | F004 | F020 | N |
| Instant noodles | F005 | F006 | N |
| Hot noodles | F006 | F007 | N |
| Noodles with black bean sauce | F007 | F008 | N |
| Cold noodles | F008 | F009 | N |
| Dumplings | F009 | F010 | N |
| Rice cakes/rice cake soup | F010 | F011 | N |
| Other rice cakes | F011 | F012 | N |
| White bread | F012 | F014 | N |
| Sweet red bean bread | F013 | F016 | N |
| Other breads | F014 | F017 | N |
| Pizza/hamburger | F015 | F019 | N |
| Cereals | F016 | F013 | N |
| Cake/chocolate pie | F017 | F018 | N |
| Snacks | F018 | F021 | N |
| Candies/chocolate | F019 | F022 | N |
| Butter | F020 | F015 | N |
| Potatoes | F021 | F029 | N |
| Sweet potatoes | F022 | F030 | N |
| Stir-fried noodles and vegetables | F023 | F031 | N |
| Beans/beans cooked in soy sauce | F024 | F024 | N |
| Tofu | F025 | F027 | N |
| Bean paste/bean paste soup | F026 | F025 | N |
| Starch jelly | F027 | F028 | N |
| Peanuts/almonds/pine nuts | F028 | F023 | N |
| Cabbage kimchi | F029 | F032 | N |
| Radish kimchi | F030 | F033 | N |
| Watery radish kimchi | F031 | F034 | N |
| Other kimchi | F032 | F035 | N |
| Green peppers | F033 | F054 | N |
| Green pepper leaves | F034 | F049 | N |
| Spinach | F035 | F039 | N |
| Lettuce | F036 | F040 | N |
| Perilla leaves | F037 | F041 | N |
| Chives/watercress | F038 | F050 | N |
| Other green vegetables | F039 | F043 | N |
| Radish | F040 | F037 | N |
| Bellflower root/Deodeck | F041 | F044 | N |
| Onion | F042 | F053 | N |
| Napa cabbage/napa cabbage soup | F043 | F038 | N |
| Cucumber | F044 | F051 | N |
| Bean sprouts/mung bean sprouts | F045 | F045 | N |
| Carrot/carrot juice | F046 | F052 | N |
| Pumpkin/kabocha squash | F047 | F056 | N |
| Zucchini | F048 | F055 | N |
| Vegetable juice | F049 | - | Excluded from visit 3 |
| Bracken/sweet potato stems | F050 | F046 | N |
| Pickled vegetables | F051 | F036 | N |
| Oyster mushrooms | F052 | F047 | N |
| Other mushrooms | F053 | F048 | N |
| Persimmon/dried persimmon | F054 | F100 | N |
| Tangerine | F055 | F101 | N |
| Oriental melon/melon | F056 | F096 | N |
| Banana | F057 | F099 | N |
| Pear | F058 | F102 | N |
| Apple | F059 | F103 | N |
| Orange/orange juice | F060 | F104 | N |
| Watermelon | F061 | F097 | N |
| Peach/plum | F062 | F098 | N |
| Strawberry | F063 | F095 | N |
| Grape/grape juice | F064 | F105 | N |
| Tomato/tomato juice/tomato sauce | F065 | F106 | N |
| Dog meat | F066 | F063 | N |
| Chicken | F067 | F064 | N |
| Grilled pork | F068 | F058 | N |
| Pork belly | F069 | F057 | N |
| Steamed pork | F070 | F059 | N |
| Processed meat | F071 | F060 | N |
| Steak/grilled beef | F072 | F062 | N |
| Beef soup/steamed beef | F073 | F066 | N |
| Organ meat | F074 | F061 | N |
| Eggs/quail eggs | F075 | F026 | N |
| Sashimi | F076 | F067 | N |
| Belt fish | F077 | F069 | N |
| Eel | F078 | F070 | N |
| Yellow croaker/sea bream/sole | F079 | F071 | N |
| Alaska pollack/frozen pollack/dried pollack | F080 | F072 | N |
| Bluefish (mackerel/pacific saury) | F081 | F068 | N |
| Anchovy/stir-fried anchovy | F082 | F074 | N |
| Squid/dried squid/octopus | F083 | F083 | N |
| Canned tuna | F084 | F075 | N |
| Fishcakes | F085 | F081 | N |
| Crab/marinated crab | F086 | F079 | N |
| Clam/sea snail | F087 | F077 | N |
| Oyster | F088 | F078 | N |
| Shrimp | F089 | F080 | N |
| Salted shrimp/salted fish | F090 | F076 | N |
| Laver | F091 | F082 | N |
| Kelp/seaweed | F092 | F083 | N |
| Milk | F093 | F084 | N |
| Yogurt/Yoplait | F094 | F085 | N |
| Ice cream | F095 | F086 | N |
| Cheese | F096 | F087 | N |
| Soft drinks (coke/cider) | F097 | F093 | N |
| Coffee | F098 | F089 | N |
| Sugars (added to tea or coffee) | F099 | F090 | N |
| Cream (added to tea or coffee) | F100 | F091 | N |
| Green tea | F101 | F092 | N |
| Soybean milk | F102 | F088 | N |
| Other beverages | F103 | F094 | N |
| - | - | F002/F004 | Rice with beans |
| - | - | F042 | Vegetable Ssam/vegetable salad |
| - | - | F065 | Tang (Ox bone soup, beef bone soup, short rib soup, ox knee soup) |

Abbreviations: KoGES, Korean genome and epidemiology study; FFQ, food frequency questionnaire; F, food item; N, No.
