# Supplementary material for: Positive association of unhealthy plant-based diets with the incidence of abdominal obesity in Korea: a comparison of baseline, most recent, and cumulative average diets
Source: Epidemiol Health. 2022 Aug 2;44:e2022063. doi: 10.4178/epih.e2022063 (PMC9754918; doi:10.4178/epih.e2022063)
Supplement: Supplementary Material 4 — Food items constituting the 17 food groups using the KoGES_Ansan Ansung Study [file epih-44-e2022063-suppl4.docx]

**Online supplementary material**

This appendix is a part of the original submission and has been peer reviewed.

Supplement to: Jung S and Park S. Positive association of unhealthy plant-based diets with the incidence of abdominal obesity: a comparison of baseline, most recent, and cumulative average diets

**Supplementary Material 1. Participant flow chart**

**Supplementary Material 2. Food items listed in the FFQ with 103 items and the FFQ with 106 items in the KoGES_Ansan Ansung Study**

**Supplementary Material 3. Illustration of the three approaches for analyzing repeated dietary measurements in the KoGES Ansan and Ansung Study**

**Supplementary Material 4. Food items constituting the 17 food groups using the KoGES_Ansan Ansung Study**

**Supplementary Material 5. Age- and sex-adjusted nutritional characteristics of the study participants according to 3 different plant-based diet indices (n=6054)**

**Supplementary Material 6. Age- and sex-adjusted food group intakes of the study participants according to 3 different plant-based diet indices (n=6054)**

**Supplementary Material 7. Adjusted HRs and 95% CIs for incident abdominal obesity according to the continuous uPDIs using restricted cubic splines**

**Supplementary Material 8. Hazard ratios (HRs) and 95% confidence intervals (CIs) for abdominal obesity according to the plant-based diet indices with salted vegetables categorized into the healthy plant food group (n=6054)**

**Supplementary Material 9. Hazard ratios (HRs) and 95% confidence intervals (CIs) for abdominal obesity according to plant-based diet indices after excluding incident cases of abdominal obesity occurring within the first two follow-up years (n=5538)**

**Supplementary Material 10. Hazard ratios (HRs) and 95% confidence intervals (CIs) for abdominal obesity according to the plant-based diet indices after excluding incident cases of hypertension, T2DM, dyslipidemia, and general obesity occurring before the development of abdominal obesity during the follow-up (n=5656)**

**Supplementary Material 4. Food items constituting the 17 food groups using the KoGES_Ansan Ansung Study**

| Food groups | Food items | PDI | hPDI | uPDI |
| --- | --- | --- | --- | --- |
| **Healthy plant foods** |  |  |  |  |
| Whole grains | Barley rice, mixed grain rice | + | + | - |
| Fruits | Persimmon/dried persimmon, tangerine, oriental melon/melon, banana, pear, apple, orange/orange juice, watermelon, peach/plum, strawberry, grape/grape juice | + | + | - |
| Vegetables | Sweet potatoes, green peppers, green pepper leaves, spinach, lettuce, perilla leaves, chives/watercress, other green vegetables, radish, bellflower root/Deodeck, onion, napa cabbage/napa cabbage soup, cucumber, bean sprouts/mung bean sprouts, carrot/carrot juice, pumpkin/kabocha squash, zucchini, vegetable juice, bracken/sweet potato stems, oyster mushrooms, other mushrooms, tomato/tomato juice/tomato sauce, laver, kelp/seaweed | + | + | - |
| Nuts | Peanuts/almonds/pine nuts | + | + | - |
| Legumes | Beans/beans cooked in soy sauce, tofu, soybean milk | + | + | - |
| Tea & Coffee | Green tea, coffee | + | + | - |
| **Less healthy plant foods** |  |  |  |  |
| Salty plant foods | Bean paste/bean paste soup, cabbage kimchi, radish kimchi, watery radish kimchi, other kimchi, pickled vegetables | + | - | + |
| Refined grains | White rice, roasted grains powder, instant noodles, hot noodles, noodles with black bean sauce, cold noodles, rice cakes/rice cake soup, other rice cakes, white bread, other breads, cereals, stir-fried noodles and vegetables, starch jelly | + | - | + |
| Potatoes | Potatoes | + | - | + |
| Sugar-sweetened beverages | Soft drinks (coke/cider), other beverages | + | - | + |
| Sweets and Desserts | Sweet red bean bread, cake/chocolate pie, snacks, candies/chocolate, sugars (added to tea or coffee) | + | - | + |
| **Animal foods** |  |  |  |  |
| Animal fat | Butter, cream (added to tea or coffee) | - | - | - |
| Dairy | Milk, ice cream, yogurt/Yoplait, cheese | - | - | - |
| Eggs | Eggs/quail eggs | - | - | - |
| Fish or Seafood | Sashimi, belt fish, eel, yellow croaker/sea bream/sole, Alaska pollack/frozen pollack/dried pollack, bluefish (mackerel/pacific saury), anchovy/stir-fried anchovy, squid/dried squid/octopus, canned tuna, fishcakes, crab/marinated crab, clam/sea snail, oyster, shrimp, salted shrimp/salted fish | - | - | - |
| Meat | Dog meat, chicken, grilled pork, pork belly, steamed pork, processed meat, steak/grilled beef, beef soup/steamed beef, organ meat | - | - | - |
| Misc. animal-based foods | Dumplings, pizza/hamburger | - | - | - |

Abbreviations: KoGES, Korean genome and epidemiology study; PDI, plant-based diet index; hPDI, healthy plant-based diet index; uPDI, unhealthy plant-based diet index.

+: Positive scores indicate that participants who were in the highest quintile of a food group received a score of 5.

-: Reverse scores indicate that participants who were in the lowest quintile of a food group received a score of 5.
