# Supplementary material for: Positive association of unhealthy plant-based diets with the incidence of abdominal obesity in Korea: a comparison of baseline, most recent, and cumulative average diets
Source: Epidemiol Health. 2022 Aug 2;44:e2022063. doi: 10.4178/epih.e2022063 (PMC9754918; doi:10.4178/epih.e2022063)
Supplement: Supplementary Material 5 — Age- and sex-adjusted nutritional characteristics of the study participants according to 3 different plant-based diet indices (n=6054) [file epih-44-e2022063-suppl5.docx]

**Online supplementary material**

This appendix is a part of the original submission and has been peer reviewed.

Supplement to: Jung S and Park S. Positive association of unhealthy plant-based diets with the incidence of abdominal obesity: a comparison of baseline, most recent, and cumulative average diets

**Supplementary Material 1. Participant flow chart**

**Supplementary Material 2. Food items listed in the FFQ with 103 items and the FFQ with 106 items in the KoGES_Ansan Ansung Study**

**Supplementary Material 3. Illustration of the three approaches for analyzing repeated dietary measurements in the KoGES Ansan and Ansung Study**

**Supplementary Material 4. Food items constituting the 17 food groups using the KoGES_Ansan Ansung Study**

**Supplementary Material 5. Age- and sex-adjusted nutritional characteristics of the study participants according to 3 different plant-based diet indices (n=6054)**

**Supplementary Material 6. Age- and sex-adjusted food group intakes of the study participants according to 3 different plant-based diet indices (n=6054)**

**Supplementary Material 7. Adjusted HRs and 95% CIs for incident abdominal obesity according to the continuous uPDIs using restricted cubic splines**

**Supplementary Material 8. Hazard ratios (HRs) and 95% confidence intervals (CIs) for abdominal obesity according to the plant-based diet indices with salted vegetables categorized into the healthy plant food group (n=6054)**

**Supplementary Material 9. Hazard ratios (HRs) and 95% confidence intervals (CIs) for abdominal obesity according to plant-based diet indices after excluding incident cases of abdominal obesity occurring within the first two follow-up years (n=5538)**

**Supplementary Material 10. Hazard ratios (HRs) and 95% confidence intervals (CIs) for abdominal obesity according to the plant-based diet indices after excluding incident cases of hypertension, T2DM, dyslipidemia, and general obesity occurring before the development of abdominal obesity during the follow-up (n=5656)**

**Supplementary Material 5. Age- and sex-adjusted nutritional characteristics of the study participants according to 3 different plant-based diet indices (n=6054)^1^**

|  | **Plant-based diet index** | | | ***P* for trend^2^** | **Healthy plant-based diet index** | | | ***P* for trend^2^** | **Unhealthy plant-based diet index** | | | ***P* for trend^2^** |
| --- | --- | --- | --- | --- | --- | --- | --- | --- | --- | --- | --- | --- |
|  | **Q1** | **Q3** | **Q5** |  | **Q1** | **Q3** | **Q5** |  | **Q1** | **Q3** | **Q5** |  |
| **Baseline diet only** |  |  |  |  |  |  |  |  |  |  |  |  |
| Protein (g/d) | 64.1 ± 0.3 | 61.7 ± 0.3 | 59.5 ± 0.3 | <.0001 | 63.4 ± 0.3 | 62.3 ± 0.3 | 59.7 ± 0.3 | <.0001 | 69.5 ± 0.3 | 62.3 ± 0.3 | 53.2 ± 0.3 | <.0001 |
| Fat (g/d) | 33.9 ± 0.3 | 29.7 ± 0.3 | 26.0 ± 0.3 | <.0001 | 33.5 ± 0.3 | 30.3 ± 0.3 | 25.5 ± 0.3 | <.0001 | 35.7 ± 0.2 | 29.8 ± 0.3 | 23.5 ± 0.2 | <.0001 |
| Carbohydrate (g/d) | 309.8 ± 0.9 | 322.2 ± 0.8 | 336.8 ± 0.9 | <.0001 | 315.5 ± 0.9 | 321.1 ± 0.9 | 332.4 ± 0.9 | <.0001 | 305.9 ± 0.8 | 322.7 ± 0.9 | 338.2 ± 0.8 | <.0001 |
| Calcium (mg/d) | 433.8 ± 5.0 | 445.7 ± 4.7 | 459.8 ± 5.0 | 0.003 | 465.0 ± 4.9 | 454.0 ± 5.2 | 416.6 ± 4.9 | <.0001 | 542.1 ± 4.5 | 448.0 ± 4.8 | 352.4 ± 4.5 | <.0001 |
| Phosphorous (mg/d) | 955.7 ± 4.7 | 959.6 ± 4.4 | 959.5 ± 4.7 | 0.86 | 953.5 ± 4.6 | 964.0 ± 4.9 | 959.7 ± 4.6 | 0.25 | 1080 ± 3.9 | 967.2 ± 4.2 | 824.8 ± 3.9 | <.0001 |
| Iron (mg/d) | 9.2 ± 0.1 | 10.0 ± 0.1 | 11.0 ± 0.1 | <.0001 | 9.4 ± 0.1 | 10.1 ± 0.1 | 10.7 ± 0.1 | <.0001 | 11.8 ± 0.1 | 10.2 ± 0.1 | 8.2 ± 0.1 | <.0001 |
| Potassium (mg/d) | 2070 ± 17 | 2336 ± 16 | 2642 ± 17 | <.0001 | 2271 ± 18 | 2347 ± 19 | 2424 ± 18 | <.0001 | 2682 ± 16 | 2369 ± 18 | 2013 ± 16 | <.0001 |
| Sodium (mg/d) | 2544 ± 35 | 3028 ± 33 | 3518 ± 35 | <.0001 | 3292 ± 35 | 3009 ± 38 | 2732 ± 35 | <.0001 | 2910 ± 35 | 3034 ± 38 | 3135 ± 35 | <.0001 |
| Vitamin B_1_ (mg/d) | 1.15 ± 0.01 | 1.15 ± 0.01 | 1.18 ± 0.01 | 0.01 | 1.14 ± 0.01 | 1.16 ± 0.01 | 1.17 ± 0.01 | 0.01 | 1.26 ± 0.01 | 1.17 ± 0.01 | 1.03 ± 0.01 | <.0001 |
| Vitamin B_2_ (mg/d) | 0.97 ± 0.01 | 0.93 ± 0.01 | 0.91 ± 0.01 | <.0001 | 0.99 ± 0.01 | 0.95 ± 0.01 | 0.88 ± 0.01 | <.0001 | 1.09 ± 0.01 | 0.94 ± 0.01 | 0.79 ± 0.01 | <.0001 |
| Niacin (mg/d) | 14.4 ± 0.1 | 14.6 ± 0.1 | 14.9 ± 0.1 | <.0001 | 14.8 ± 0.1 | 14.6 ± 0.1 | 14.3 ± 0.1 | <.0001 | 16.4 ± 0.1 | 14.7 ± 0.1 | 12.7 ± 0.1 | <.0001 |
| Vitamin C (mg/d) | 87.9 ± 1.7 | 112.4 ± 1.5 | 142.0 ± 1.7 | <.0001 | 100.9 ± 1.7 | 114.3 ± 1.8 | 127.5 ± 1.7 | <.0001 | 129.7 ± 1.7 | 115.5 ± 1.8 | 99.8 ± 1.7 | <.0001 |
| Zinc (mg/d) | 8.4 ± 0.1 | 8.1 ± 0.1 | 7.9 ± 0.1 | <.0001 | 8.3 ± 0.1 | 8.2 ± 0.1 | 8.0 ± 0.1 | <.0001 | 9.1 ± 0.1 | 8.2 ± 0.1 | 7.1 ± 0.1 | <.0001 |
| Vitamin B_6_ (mg/d) | 1.56 ± 0.01 | 1.66 ± 0.01 | 1.79 ± 0.01 | <.0001 | 1.67 ± 0.01 | 1.68 ± 0.01 | 1.67 ± 0.01 | 0.78 | 1.82 ± 0.01 | 1.69 ± 0.01 | 1.50 ± 0.01 | <.0001 |
| Folate (µg/d) | 190.5 ± 2.1 | 228.0 ± 2.0 | 266.8 ± 2.1 | <.0001 | 216.0 ± 2.2 | 227.0 ± 2.3 | 242.7 ± 2.2 | <.0001 | 255.7 ± 2.1 | 230.3 ± 2.2 | 199.0 ± 2.1 | <.0001 |
| Retinol (µg/d) | 83.8 ± 1.3 | 62.3 ± 1.2 | 42.8 ± 1.3 | <.0001 | 81.3 ± 1.3 | 66.4 ± 1.3 | 44.3 ± 1.3 | <.0001 | 89.9 ± 1.2 | 61.5 ± 1.3 | 39.7 ± 1.2 | <.0001 |
| Beta-carotene (µg/d) | 1932 ± 51 | 2544 ± 47 | 3167 ± 51 | <.0001 | 2424 ± 51 | 2514 ± 54 | 2606 ± 51 | 0.01 | 2972 ± 50 | 2491 ± 54 | 2211 ± 50 | <.0001 |
| Fiber (g/d) | 5.2 ± 0.1 | 6.5 ± 0.1 | 7.9 ± 0.1 | <.0001 | 5.7 ± 0.1 | 6.4 ± 0.1 | 7.4 ± 0.1 | <.0001 | 7.0 ± 0.1 | 6.6 ± 0.1 | 6.0 ± 0.1 | <.0001 |
| Vitamin E (mg/d) | 7.9 ± 0.1 | 8.6 ± 0.1 | 9.3 ± 0.1 | <.0001 | 8.5 ± 0.1 | 8.6 ± 0.1 | 8.4 ± 0.1 | 0.25 | 9.8 ± 0.1 | 8.6 ± 0.1 | 7.3 ± 0.1 | <.0001 |
| Cholesterol (mg/d) | 204.0 ± 2.6 | 160.4 ± 2.4 | 118.5 ± 2.6 | <.0001 | 203.8 ± 2.5 | 165.5 ± 2.7 | 113.7 ± 2.5 | <.0001 | 222.7 ± 2.4 | 158.7 ± 2.5 | 100.9 ± 2.4 | <.0001 |
| **Most recent diet** |  |  |  |  |  |  |  |  |  |  |  |  |
| Protein (g/d) | 59.1 ± 0.3 | 58.1 ± 0.3 | 56.6 ± 0.3 | <.0001 | 60.1 ± 0.3 | 58.0 ± 0.3 | 55.6 ± 0.3 | <.0001 | 64.5 ± 0.3 | 58.3 ± 0.3 | 50.3 ± 0.3 | <.0001 |
| Fat (g/d) | 30.0 ± 0.3 | 27.4 ± 0.3 | 24.2 ± 0.3 | <.0001 | 31.8 ± 0.3 | 27.3 ± 0.3 | 22.4 ± 0.3 | <.0001 | 32.4 ± 0.3 | 27.3 ± 0.3 | 21.7 ± 0.3 | <.0001 |
| Carbohydrate (g/d) | 303.9 ± 1.0 | 314.5 ± 0.9 | 326.9 ± 1.0 | <.0001 | 303.1 ± 0.9 | 313.3 ± 1.0 | 327.4 ± 1.0 | <.0001 | 297.2 ± 0.9 | 316.1 ± 1.0 | 327.0 ± 0.9 | <.0001 |
| Calcium (mg/d) | 382.8 ± 5.1 | 425.3 ± 4.7 | 449.0 ± 5.2 | <.0001 | 448.0 ± 5.1 | 417.1 ± 5.1 | 395.1 ± 5.1 | <.0001 | 506.4 ± 4.6 | 422.6 ± 5.1 | 334.2 ± 4.8 | <.0001 |
| Phosphorous (mg/d) | 873.9 ± 5.0 | 898.7 ± 4.6 | 914.2 ± 5.1 | <.0001 | 907.8 ± 5.0 | 893.7 ± 5.0 | 886.4 ± 5.0 | 0.0004 | 999.0 ± 4.3 | 902.3 ± 4.8 | 777.1 ± 4.4 | <.0001 |
| Iron (mg/d) | 8.5 ± 0.1 | 9.6 ± 0.1 | 10.5 ± 0.1 | <.0001 | 9.3 ± 0.1 | 9.5 ± 0.1 | 9.8 ± 0.1 | <.0001 | 11.1 ± 0.1 | 9.6 ± 0.1 | 7.9 ± 0.1 | <.0001 |
| Potassium (mg/d) | 1913 ± 19 | 2259 ± 17 | 2590 ± 19 | <.0001 | 2253 ± 20 | 2233 ± 20 | 2281 ± 20 | 0.34 | 2639 ± 18 | 2275 ± 19 | 1867 ± 18 | <.0001 |
| Sodium (mg/d) | 2262 ± 36 | 2773 ± 33 | 3256 ± 36 | <.0001 | 3089 ± 37 | 2728 ± 37 | 2471 ± 37 | <.0001 | 2712 ± 36 | 2804 ± 39 | 2782 ± 37 | 0.29 |
| Vitamin B_1_ (mg/d) | 1.02 ± 0.01 | 1.03 ± 0.01 | 1.06 ± 0.01 | <.0001 | 1.06 ± 0.01 | 1.03 ± 0.01 | 1.02 ± 0.01 | <.0001 | 1.10 ± 0.01 | 1.05 ± 0.01 | 0.96 ± 0.01 | <.0001 |
| Vitamin B_2_ (mg/d) | 0.87 ± 0.01 | 0.89 ± 0.01 | 0.89 ± 0.01 | 0.02 | 0.94 ± 0.01 | 0.88 ± 0.01 | 0.83 ± 0.01 | <.0001 | 1.03 ± 0.01 | 0.88 ± 0.01 | 0.74 ± 0.01 | <.0001 |
| Niacin (mg/d) | 13.4 ± 0.1 | 13.8 ± 0.1 | 14.3 ± 0.1 | <.0001 | 14.3 ± 0.1 | 13.8 ± 0.1 | 13.3 ± 0.1 | <.0001 | 15.4 ± 0.1 | 13.9 ± 0.1 | 12.0 ± 0.1 | <.0001 |
| Vitamin C (mg/d) | 76.6 ± 1.5 | 104.7 ± 1.4 | 132.9 ± 1.5 | <.0001 | 97.7 ± 1.6 | 102.9 ± 1.6 | 114.4 ± 1.6 | <.0001 | 123.8 ± 1.5 | 107.4 ± 1.6 | 86.0 ± 1.5 | <.0001 |
| Zinc (mg/d) | 7.9 ± 0.1 | 7.7 ± 0.1 | 7.5 ± 0.1 | <.0001 | 7.9 ± 0.1 | 7.7 ± 0.1 | 7.6 ± 0.1 | <.0001 | 8.4 ± 0.1 | 7.7 ± 0.1 | 6.8 ± 0.1 | <.0001 |
| Vitamin B_6_ (mg/d) | 1.44 ± 0.01 | 1.57 ± 0.01 | 1.70 ± 0.01 | <.0001 | 1.60 ± 0.01 | 1.56 ± 0.01 | 1.53 ± 0.01 | <.0001 | 1.69 ± 0.01 | 1.59 ± 0.01 | 1.40 ± 0.01 | <.0001 |
| Folate (µg/d) | 172.3 ± 2.3 | 215.6 ± 2.1 | 255.3 ± 2.3 | <.0001 | 212.7 ± 2.4 | 211.3 ± 2.4 | 217.7 ± 2.4 | 0.11 | 238.1 ± 2.2 | 217.6 ± 2.5 | 189.3 ± 2.3 | <.0001 |
| Retinol (µg/d) | 71.0 ± 1.2 | 58.3 ± 1.1 | 42.6 ± 1.2 | <.0001 | 75.6 ± 1.2 | 56.6 ± 1.2 | 41.3 ± 1.2 | <.0001 | 80.5 ± 1.1 | 57.4 ± 1.2 | 36.2 ± 1.2 | <.0001 |
| Beta-carotene (µg/d) | 1721 ± 47 | 2420 ± 43 | 2928 ± 47 | <.0001 | 2376 ± 47 | 2304 ± 48 | 2331 ± 48 | 0.60 | 2695 ± 45 | 2378 ± 50 | 2046 ± 47 | <.0001 |
| Fiber (g/d) | 4.8 ± 0.1 | 6.0 ± 0.1 | 7.3 ± 0.1 | <.0001 | 5.6 ± 0.1 | 5.9 ± 0.1 | 6.5 ± 0.1 | <.0001 | 6.5 ± 0.1 | 6.1 ± 0.1 | 5.5 ± 0.1 | <.0001 |
| Vitamin E (mg/d) | 7.1 ± 0.1 | 8.0 ± 0.1 | 8.8 ± 0.1 | <.0001 | 8.1 ± 0.1 | 7.9 ± 0.1 | 7.8 ± 0.1 | <.0001 | 9.3 ± 0.1 | 8.0 ± 0.1 | 6.7 ± 0.1 | <.0001 |
| Cholesterol (mg/d) | 179.5 ± 2.6 | 149.1 ± 2.4 | 109.5 ± 2.6 | <.0001 | 191.9 ± 2.5 | 145.3 ± 2.5 | 100.4 ± 2.5 | <.0001 | 204.8 ± 2.3 | 144.3 ± 2.5 | 88.9 ± 2.4 | <.0001 |
| **Cumulative average** |  |  |  |  |  |  |  |  |  |  |  |  |
| Protein (g/d) | 61.5 ± 0.3 | 60.0 ± 0.2 | 58.6 ± 0.3 | <.0001 | 62.0 ± 0.3 | 59.8 ± 0.3 | 58.4 ± 0.3 | <.0001 | 66.5 ± 0.2 | 60.0 ± 0.2 | 52.5 ± 0.2 | <.0001 |
| Fat (g/d) | 31.7 ± 0.2 | 28.6 ± 0.2 | 25.6 ± 0.2 | <.0001 | 32.6 ± 0.2 | 28.6 ± 0.2 | 24.6 ± 0.2 | <.0001 | 33.4 ± 0.2 | 28.6 ± 0.2 | 23.0 ± 0.2 | <.0001 |
| Carbohydrate (g/d) | 308.8 ± 0.8 | 318.0 ± 0.7 | 330.0 ± 0.8 | <.0001 | 310.9 ± 0.8 | 317.8 ± 0.8 | 329.1 ± 0.8 | <.0001 | 304.7 ± 0.8 | 319.0 ± 0.8 | 331.7 ± 0.8 | <.0001 |
| Calcium (mg/d) | 414.2 ± 4.3 | 437.3 ± 4.0 | 450.5 ± 4.3 | <.0001 | 455.0 ± 4.4 | 435.9 ± 4.5 | 415.9 ± 4.4 | <.0001 | 515.1 ± 4.1 | 434.6 ± 4.2 | 347.9 ± 4.1 | <.0001 |
| Phosphorous (mg/d) | 918.7 ± 4.1 | 929.5 ± 3.9 | 939.5 ± 4.1 | 0.0002 | 936.0 ± 4.2 | 925.7 ± 4.3 | 934.5 ± 4.2 | 0.92 | 1031.7 ± 3.6 | 930.8 ± 3.7 | 811.5 ± 3.6 | <.0001 |
| Iron (mg/d) | 9.0 ± 0.1 | 9.8 ± 0.1 | 10.7 ± 0.1 | <.0001 | 9.4 ± 0.1 | 9.8 ± 0.1 | 10.4 ± 0.1 | <.0001 | 11.3 ± 0.1 | 9.8 ± 0.1 | 8.2 ± 0.1 | <.0001 |
| Potassium (mg/d) | 2048 ± 15 | 2295 ± 14 | 2570 ± 15 | <.0001 | 2269 ± 16 | 2291 ± 17 | 2380 ± 16 | <.0001 | 2627 ± 15 | 2300 ± 15 | 1974 ± 15 | <.0001 |
| Sodium (mg/d) | 2514 ± 30 | 2894 ± 28 | 3343 ± 30 | <.0001 | 3154 ± 31 | 2877 ± 32 | 2710 ± 31 | <.0001 | 2829 ± 31 | 2869 ± 32 | 2998 ± 32 | 0.0004 |
| Vitamin B_1_ (mg/d) | 1.10 ± 0.01 | 1.09 ± 0.01 | 1.12 ± 0.01 | 0.001 | 1.11 ± 0.01 | 1.09 ± 0.01 | 1.11 ± 0.01 | 0.84 | 1.18 ± 0.01 | 1.10 ± 0.01 | 1.01 ± 0.01 | <.0001 |
| Vitamin B_2_ (mg/d) | 0.92 ± 0.01 | 0.91 ± .01 | 0.90 ± 0.01 | 0.001 | 0.96 ± 0.01 | 0.92 ± 0.01 | 0.87 ± 0.01 | <.0001 | 1.05 ± 0.01 | 0.91 ± 0.01 | 0.77 ± 0.01 | <.0001 |
| Niacin (mg/d) | 14.0 ± 0.1 | 14.2 ± 0.1 | 14.6 ± 0.1 | <.0001 | 14.7 ± 0.1 | 14.1 ± 0.1 | 13.9 ± 0.1 | <.0001 | 15.8 ± 0.1 | 14.2 ± 0.1 | 12.5 ± 0.1 | <.0001 |
| Vitamin C (mg/d) | 87.7 ± 1.4 | 108.6 ± 1.3 | 132.7 ± 1.4 | <.0001 | 100.7 ± 1.4 | 108.6 ± 1.5 | 122.3 ± 1.4 | <.0001 | 125.1 ± 1.4 | 109.3 ± 1.4 | 95.4 ± 1.4 | <.0001 |
| Zinc (mg/d) | 8.2 ± 0.0 | 7.9 ± 0.0 | 7.7 ± 0.0 | <.0001 | 8.1 ± 0.0 | 7.9 ± 0.0 | 7.9 ± 0.0 | 0.001 | 8.8 ± 0.0 | 7.9 ± 0.0 | 7.0 ± 0.0 | <.0001 |
| Vitamin B_6_ (mg/d) | 1.53 ± 0.01 | 1.61 ± 0.01 | 1.73 ± 0.01 | <.0001 | 1.64 ± 0.01 | 1.61 ± 0.01 | 1.63 ± 0.01 | 0.40 | 1.75 ± 0.01 | 1.62 ± 0.01 | 1.47 ± 0.01 | <.0001 |
| Folate (µg/d) | 188.5 ± 1.8 | 221.0 ± 1.7 | 254.9 ± 1.8 | <.0001 | 213.8 ± 1.9 | 218.9 ± 2.0 | 234.2 ± 1.9 | <.0001 | 244.7 ± 1.9 | 220.6 ± 1.9 | 197.3 ± 1.9 | <.0001 |
| Retinol (µg/d) | 75.5 ± 1.1 | 61.1 ± 1.0 | 44.9 ± 1.1 | <.0001 | 76.9 ± 1.1 | 62.0 ± 1.1 | 44.8 ± 1.1 | <.0001 | 82.2 ± 1.0 | 60.4 ± 1.0 | 38.9 ± 1.0 | <.0001 |
| Beta-carotene (µg/d) | 1944 ± 41 | 2474 ± 38 | 2924 ± 41 | <.0001 | 2380 ± 42 | 2392 ± 43 | 2547 ± 43 | 0.003 | 2791 ± 42 | 2390 ± 43 | 2193 ± 42 | <.0001 |
| Fiber (g/d) | 5.2 ± 0.0 | 6.2 ± 0.0 | 7.4 ± 0.0 | <.0001 | 5.7 ± 0.1 | 6.2 ± 0.1 | 7.0 ± 0.1 | <.0001 | 6.7 ± 0.1 | 6.2 ± 0.1 | 5.8 ± 0.1 | <.0001 |
| Vitamin E (mg/d) | 7.7 ± 0.1 | 8.3 ± 0.1 | 8.9 ± 0.1 | <.0001 | 8.4 ± 0.1 | 8.2 ± 0.1 | 8.2 ± 0.1 | 0.03 | 9.4 ± 0.1 | 8.2 ± 0.1 | 7.1 ± 0.1 | <.0001 |
| Cholesterol (mg/d) | 188.4 ± 2.2 | 155.9 ± 2.0 | 119.8 ± 2.2 | <.0001 | 196.1 ± 2.2 | 155.1 ± 2.2 | 115.3 ± 2.2 | <.0001 | 207.9 ± 2.1 | 152.7 ± 2.1 | 99.5 ± 2.1 | <.0001 |

Abbreviation: Q, quintile.

^1^ All nutrient values were adjusted for total energy intake using the residual method. Estimated mean and standard error were obtained using the linear regression model after adjusting for age and sex.

^2^ *P* for linear trend was determined by treating the median value of each group as a continuous variable using a general linear model.
