# Supplementary material for: Positive association of unhealthy plant-based diets with the incidence of abdominal obesity in Korea: a comparison of baseline, most recent, and cumulative average diets
Source: Epidemiol Health. 2022 Aug 2;44:e2022063. doi: 10.4178/epih.e2022063 (PMC9754918; doi:10.4178/epih.e2022063)
Supplement: Supplementary Material 6 — Age- and sex-adjusted food group intakes of the study participants according to 3 different plant-based diet indices (n=6054) [file epih-44-e2022063-suppl6.docx]

**Online supplementary material**

This appendix is a part of the original submission and has been peer reviewed.

Supplement to: Jung S and Park S. Positive association of unhealthy plant-based diets with the incidence of abdominal obesity: a comparison of baseline, most recent, and cumulative average diets

**Supplementary Material 1. Participant flow chart**

**Supplementary Material 2. Food items listed in the FFQ with 103 items and the FFQ with 106 items in the KoGES_Ansan Ansung Study**

**Supplementary Material 3. Illustration of the three approaches for analyzing repeated dietary measurements in the KoGES Ansan and Ansung Study**

**Supplementary Material 4. Food items constituting the 17 food groups using the KoGES_Ansan Ansung Study**

**Supplementary Material 5. Age- and sex-adjusted nutritional characteristics of the study participants according to 3 different plant-based diet indices (n=6054)**

**Supplementary Material 6. Age- and sex-adjusted food group intakes of the study participants according to 3 different plant-based diet indices (n=6054)**

**Supplementary Material 7. Adjusted HRs and 95% CIs for incident abdominal obesity according to the continuous uPDIs using restricted cubic splines**

**Supplementary Material 8. Hazard ratios (HRs) and 95% confidence intervals (CIs) for abdominal obesity according to the plant-based diet indices with salted vegetables categorized into the healthy plant food group (n=6054)**

**Supplementary Material 9. Hazard ratios (HRs) and 95% confidence intervals (CIs) for abdominal obesity according to plant-based diet indices after excluding incident cases of abdominal obesity occurring within the first two follow-up years (n=5538)**

**Supplementary Material 10. Hazard ratios (HRs) and 95% confidence intervals (CIs) for abdominal obesity according to the plant-based diet indices after excluding incident cases of hypertension, T2DM, dyslipidemia, and general obesity occurring before the development of abdominal obesity during the follow-up (n=5656)**

**Supplementary Material 6. Age- and sex-adjusted food group intakes of the study participants according to 3 different plant-based diet indices (n=6054)^1^**

|  | **Plant-based diet index** | | | ***P* for trend^2^** | **Healthy plant-based diet index** | | | ***P* for trend^2^** | **Unhealthy plant-based diet index** | | | ***P* for trend^2^** |
| --- | --- | --- | --- | --- | --- | --- | --- | --- | --- | --- | --- | --- |
|  | **Q1** | **Q3** | **Q5** |  | **Q1** | **Q3** | **Q5** |  | **Q1** | **Q3** | **Q5** |  |
| **Baseline diet only** |  |  |  |  |  |  |  |  |  |  |  |  |
| Whole grains | 1.85 ± 0.09 | 1.87 ± 0.08 | 1.64 ± 0.09 | 0.11 | 0.56 ± 0.08 | 1.74 ± 0.09 | 3.24 ± 0.08 | <.0001 | 3.24 ± 0.08 | 1.80 ± 0.09 | 0.54 ± 0.08 | <.0001 |
| Fruits | 1.25 ± 0.04 | 1.56 ± 0.04 | 2.01 ± 0.04 | <.0001 | 1.14 ± 0.04 | 1.64 ± 0.05 | 2.06 ± 0.04 | <.0001 | 1.98 ± 0.04 | 1.64 ± 0.05 | 1.27 ± 0.04 | <.0001 |
| Vegetables | 3.17 ± 0.06 | 3.84 ± 0.06 | 4.68 ± 0.06 | <.0001 | 3.59 ± 0.06 | 3.88 ± 0.06 | 4.12 ± 0.06 | <.0001 | 4.96 ± 0.06 | 3.91 ± .06 | 2.83 ± 0.06 | <.0001 |
| Nuts | 0.03 ± 0.01 | 0.07 ± 0.01 | 0.08 ± 0.01 | <.0001 | 0.04 ± 0.01 | 0.06 ± 0.01 | 0.06 ± 0.01 | 0.06 | 0.13 ± 0.01 | 0.05 ± 0.01 | 0.01 ± 0.01 | <.0001 |
| Legumes | 0.52 ± 0.02 | 0.75 ± 0.02 | 1.14 ± 0.02 | <.0001 | 0.52 ± 0.02 | 0.77 ± 0.02 | 1.14 ± 0.02 | <.0001 | 1.09 ± 0.02 | 0.80 ± 0.02 | 0.43 ± 0.02 | <.0001 |
| Tea and coffee | 1.10 ± 0.05 | 1.46 ± 0.04 | 1.90 ± 0.05 | <.0001 | 1.67 ± 0.05 | 1.45 ± 0.05 | 1.26 ± 0.05 | <.0001 | 1.97 ± 0.04 | 1.41 ± 0.05 | 1.09 ± 0.04 | <.0001 |
| Refined grains | 2.41 ± 0.04 | 2.22 ± 0.04 | 2.18 ± 0.04 | <.0001 | 3.27 ± 0.04 | 2.20 ± 0.04 | 1.25 ± 0.04 | <.0001 | 1.34 ± 0.04 | 2.16 ± 0.04 | 3.19 ± 0.04 | <.0001 |
| Potatoes | 0.17 ± 0.01 | 0.26 ± 0.01 | 0.36 ± 0.01 | <.0001 | 0.33 ± 0.01 | 0.27 ± 0.01 | 0.18 ± 0.01 | <.0001 | 0.29 ± 0.01 | 0.25 ± 0.01 | 0.23 ± 0.01 | 0.0002 |
| Sugar-sweetened beverages | 0.17 ± 0.01 | 0.19 ± 0.01 | 0.24 ± 0.01 | 0.0002 | 0.34 ± 0.01 | 0.19 ± 0.01 | 0.08 ± 0.01 | <.0001 | 0.21 ± 0.01 | 0.20 ± 0.01 | 0.20 ± 0.01 | 0.84 |
| Sweets and desserts | 1.02 ± 0.04 | 1.25 ± 0.04 | 1.62 ± 0.04 | <.0001 | 1.76 ± 0.04 | 1.25 ± 0.04 | 0.74 ± 0.04 | <.0001 | 1.40 ± 0.04 | 1.30 ± 0.04 | 1.12 ± 0.04 | <.0001 |
| Salty vegetables | 3.04 ± 0.06 | 4.05 ± 0.06 | 5.12 ± 0.06 | <.0001 | 4.41 ± 0.06 | 3.99 ± 0.07 | 3.69 ± 0.06 | <.0001 | 3.52 ± 0.06 | 4.08 ± 0.07 | 4.63 ± 0.06 | <.0001 |
| Animal fat | 0.72 ± 0.03 | 0.82 ± 0.03 | 0.81 ± 0.03 | 0.03 | 1.21 ± 0.03 | 0.80 ± 0.03 | 0.35 ± 0.03 | <.0001 | 1.01 ± 0.03 | 0.81 ± 0.03 | 0.53 ± 0.03 | <.0001 |
| Dairy | 1.14 ± 0.03 | 0.73 ± 0.03 | 0.42 ± 0.03 | <.0001 | 1.05 ± 0.03 | 0.85 ± 0.03 | 0.45 ± 0.03 | <.0001 | 1.29 ± 0.03 | 0.70 ± 0.03 | 0.40 ± 0.03 | <.0001 |
| Eggs | 0.37 ± 0.01 | 0.25 ± 0.01 | 0.15 ± 0.01 | <.0001 | 0.37 ± 0.01 | 0.27 ± 0.01 | 0.14 ± 0.01 | <.0001 | 0.41 ± 0.01 | 0.23 ± 0.01 | 0.13 ± 0.01 | <.0001 |
| Fish | 1.31 ± 0.03 | 1.20 ± 0.03 | 1.01 ± 0.03 | <.0001 | 1.41 ± 0.03 | 1.17 ± 0.03 | 0.91 ± 0.03 | <.0001 | 1.72 ± 0.02 | 1.18 ± 0.03 | 0.66 ± 0.02 | <.0001 |
| Meat | 0.68 ± 0.01 | 0.48 ± 0.01 | 0.34 ± 0.01 | <.0001 | 0.66 ± 0.01 | 0.50 ± 0.01 | 0.34 ± 0.01 | <.0001 | 0.68 ± 0.01 | 0.49 ± 0.01 | 0.32 ± 0.01 | <.0001 |
| Miscellaneous animal foods | 0.07 ± 0.003 | 0.04 ± 0.003 | 0.02 ± 0.003 | <.0001 | 0.07 ± 0.003 | 0.04 ± 0.004 | 0.02 ± 0.003 | <.0001 | 0.08 ± 0.003 | 0.04 ± 0.004 | 0.02 ± 0.003 | <.0001 |
| **Most recent diet** |  |  |  |  |  |  |  |  |  |  |  |  |
| Whole grains | 2.20 ± 0.07 | 2.15 ± 0.07 | 2.21 ± 0.07 | 0.70 | 0.96 ± 0.07 | 2.21 ± 0.07 | 3.30 ± 0.07 | <.0001 | 3.17 ± 0.07 | 2.21 ± 0.07 | 1.11 ± 0.07 | <.0001 |
| Fruits | 1.05 ± 0.04 | 1.39 ± 0.03 | 1.80 ± 0.04 | <.0001 | 1.10 ± 0.04 | 1.39 ± 0.04 | 1.74 ± 0.04 | <.0001 | 1.84 ± 0.03 | 1.46 ± 0.04 | 0.95 ± 0.04 | <.0001 |
| Vegetables | 2.84 ± 0.06 | 3.71 ± 0.06 | 4.63 ± 0.06 | <.0001 | 3.49 ± 0.07 | 3.64 ± 0.07 | 4.02 ± 0.07 | <.0001 | 4.88 ± 0.06 | 3.69 ± 0.07 | 2.59 ± 0.06 | <.0001 |
| Nuts | 0.03 ± 0.01 | 0.07 ± 0.01 | 0.10 ± 0.01 | <.0001 | 0.05 ± 0.01 | 0.06 ± 0.01 | 0.08 ± 0.01 | 0.01 | 0.15 ± 0.01 | 0.05 ± 0.01 | 0.02 ± 0.01 | <.0001 |
| Legumes | 0.40 ± 0.02 | 0.60 ± 0.02 | 0.88 ± 0.02 | <.0001 | 0.49 ± 0.02 | 0.59 ± 0.02 | 0.82 ± 0.02 | <.0001 | 0.90 ± 0.02 | 0.60 ± 0.02 | 0.35 ± 0.02 | <.0001 |
| Tea and coffee | 1.36 ± 0.06 | 1.93 ± 0.06 | 2.55 ± 0.06 | <.0001 | 2.22 ± 0.06 | 1.97 ± 0.06 | 1.65 ± 0.06 | <.0001 | 2.81 ± 0.06 | 1.84 ± 0.06 | 1.25 ± 0.06 | <.0001 |
| Refined grains | 1.76 ± 0.05 | 1.59 ± 0.04 | 1.49 ± 0.05 | <.0001 | 2.74 ± 0.04 | 1.48 ± 0.04 | 0.79 ± 0.04 | <.0001 | 0.90 ± 0.04 | 1.54 ± 0.05 | 2.42 ± 0.04 | <.0001 |
| Potatoes | 0.14 ± 0.01 | 0.25 ± 0.01 | 0.34 ± 0.01 | <.0001 | 0.32 ± 0.01 | 0.23 ± 0.01 | 0.17 ± 0.01 | <.0001 | 0.29 ± 0.01 | 0.23 ± 0.01 | 0.21 ± 0.01 | <.0001 |
| Sugar-sweetened beverages | 0.14 ± 0.01 | 0.17 ± 0.01 | 0.23 ± 0.01 | <.0001 | 0.31 ± 0.01 | 0.17 ± 0.01 | 0.07 ± 0.01 | <.0001 | 0.17 ± 0.01 | 0.19 ± 0.01 | 0.19 ± 0.01 | 0.29 |
| Sweets and desserts | 1.08 ± 0.06 | 1.43 ± 0.05 | 1.87 ± 0.06 | <.0001 | 2.07 ± 0.05 | 1.44 ± 0.05 | 0.81 ± 0.05 | <.0001 | 1.78 ± 0.05 | 1.42 ± 0.06 | 1.10 ± 0.05 | <.0001 |
| Salty vegetables | 2.78 ± 0.06 | 3.79 ± 0.06 | 4.83 ± 0.06 | <.0001 | 4.19 ± 0.07 | 3.73 ± 0.07 | 3.44 ± 0.07 | <.0001 | 3.42 ± 0.06 | 3.85 ± 0.07 | 4.17 ± 0.07 | <.0001 |
| Animal fat | 0.90 ± 0.06 | 1.10 ± 0.05 | 1.16 ± 0.06 | <.0001 | 1.75 ± 0.05 | 1.06 ± 0.05 | 0.46 ± 0.05 | <.0001 | 1.59 ± 0.05 | 1.02 ± 0.06 | 0.63 ± 0.05 | <.0001 |
| Dairy | 0.80 ± 0.03 | 0.60 ± 0.03 | 0.37 ± 0.03 | <.0001 | 0.92 ± 0.03 | 0.61 ± 0.03 | 0.38 ± 0.03 | <.0001 | 1.11 ± 0.03 | 0.56 ± 0.03 | 0.25 ± 0.03 | <.0001 |
| Eggs | 0.35 ± 0.01 | 0.25 ± 0.01 | 0.15 ± 0.01 | <.0001 | 0.38 ± 0.01 | 0.23 ± 0.01 | 0.12 ± 0.01 | <.0001 | 0.42 ± 0.01 | 0.22 ± 0.01 | 0.10 ± 0.01 | <.0001 |
| Fish | 1.16 ± 0.03 | 1.09 ± 0.02 | 0.93 ± 0.03 | <.0001 | 1.32 ± 0.03 | 1.05 ± 0.03 | 0.83 ± 0.03 | <.0001 | 1.60 ± 0.02 | 1.03 ± 0.03 | 0.55 ± 0.02 | <.0001 |
| Meat | 0.59 ± 0.01 | 0.42 ± 0.01 | 0.29 ± 0.01 | <.0001 | 0.61 ± 0.01 | 0.43 ± 0.01 | 0.27 ± 0.01 | <.0001 | 0.58 ± 0.01 | 0.43 ± 0.01 | 0.29 ± 0.01 | <.0001 |
| Miscellaneous animal foods | 0.06 ± 0.003 | 0.04 ± 0.003 | 0.02 ± 0.003 | <.0001 | 0.07 ± 0.003 | 0.03 ± 0.003 | 0.02 ± 0.003 | <.0001 | 0.07 ± 0.003 | 0.03 ± 0.003 | 0.02 ± 0.003 | <.0001 |
| **Cumulative average** |  |  |  |  |  |  |  |  |  |  |  |  |
| Whole grains | 1.92 ± 0.07 | 2.01 ± 0.07 | 2.01 ± 0.07 | 0.47 | 0.88 ± 0.07 | 1.94 ± 0.07 | 3.22 ± 0.07 | <.0001 | 3.02 ± 0.07 | 2.10 ± 0.07 | 0.92 ± 0.07 | <.0001 |
| Fruits | 1.24 ± 0.03 | 1.47 ± 0.03 | 1.81 ± 0.03 | <.0001 | 1.20 ± 0.04 | 1.52 ± 0.04 | 1.89 ± 0.04 | <.0001 | 1.86 ± 0.04 | 1.53 ± 0.04 | 1.18 ± 0.04 | <.0001 |
| Vegetables | 3.11 ± 0.05 | 3.83 ± 0.05 | 4.49 ± 0.05 | <.0001 | 3.54 ± 0.05 | 3.75 ± 0.06 | 4.10 ± 0.06 | <.0001 | 4.78 ± 0.05 | 3.77 ± 0.05 | 2.83 ± 0.05 | <.0001 |
| Nuts | 0.04 ± 0.01 | 0.06 ± 0.0049 | 0.08 ± 0.01 | <.0001 | 0.06 ± 0.01 | 0.06 ± 0.01 | 0.07 ± 0.01 | 0.11 | 0.13 ± 0.01 | 0.05 ± 0.01 | 0.02 ± 0.01 | <.0001 |
| Legumes | 0.49 ± 0.02 | 0.67 ± 0.02 | 1.00 ± 0.02 | <.0001 | 0.52 ± 0.02 | 0.67 ± 0.02 | 0.97 ± 0.02 | <.0001 | 0.97 ± 0.02 | 0.69 ± 0.02 | 0.43 ± 0.02 | <.0001 |
| Tea and coffee | 1.30 ± 0.05 | 1.68 ± 0.04 | 2.15 ± 0.05 | <.0001 | 1.93 ± 0.05 | 1.68 ± 0.05 | 1.42 ± 0.05 | <.0001 | 2.25 ± 0.05 | 1.68 ± 0.05 | 1.19 ± 0.05 | <.0001 |
| Refined grains | 2.15 ± 0.04 | 1.90 ± 0.04 | 1.84 ± 0.04 | <.0001 | 2.85 ± 0.03 | 1.86 ± 0.04 | 1.09 ± 0.03 | <.0001 | 1.20 ± 0.04 | 1.83 ± 0.04 | 2.80 ± 0.04 | <.0001 |
| Potatoes | 0.17 ± 0.01 | 0.25 ± 0.01 | 0.33 ± 0.01 | <.0001 | 0.31 ± 0.01 | 0.24 ± 0.01 | 0.19 ± 0.01 | <.0001 | 0.28 ± 0.01 | 0.24 ± 0.01 | 0.22 ± 0.01 | <.0001 |
| Sugar-sweetened beverages | 0.17 ± 0.01 | 0.19 ± 0.01 | 0.22 ± 0.01 | <.0001 | 0.31 ± 0.01 | 0.19 ± 0.01 | 0.08 ± 0.01 | <.0001 | 0.19 ± 0.01 | 0.20 ± 0.01 | 0.19 ± 0.01 | 0.44 |
| Sweets and desserts | 1.09 ± 0.04 | 1.35 ± 0.04 | 1.70 ± 0.04 | <.0001 | 1.90 ± 0.04 | 1.38 ± 0.04 | 0.78 ± 0.04 | <.0001 | 1.51 ± 0.04 | 1.39 ± 0.04 | 1.11 ± 0.04 | <.0001 |
| Salty vegetables | 3.09 ± 0.05 | 3.90 ± 0.05 | 4.89 ± 0.05 | <.0001 | 4.22 ± 0.06 | 3.90 ± 0.06 | 3.73 ± 0.06 | <.0001 | 3.53 ± 0.06 | 3.87 ± 0.06 | 4.44 ± 0.06 | <.0001 |
| Animal fat | 0.81 ± 0.04 | 0.97 ± 0.04 | 0.95 ± 0.04 | 0.001 | 1.46 ± 0.04 | 0.94 ± 0.04 | 0.44 ± 0.04 | <.0001 | 1.21 ± 0.04 | 0.97 ± 0.04 | 0.60 ± 0.04 | <.0001 |
| Dairy | 0.95 ± 0.03 | 0.68 ± 0.03 | 0.44 ± 0.03 | <.0001 | 0.94 ± 0.03 | 0.74 ± 0.03 | 0.46 ± 0.03 | <.0001 | 1.14 ± 0.03 | 0.67 ± 0.03 | 0.36 ± 0.03 | <.0001 |
| Eggs | 0.35 ± 0.01 | 0.25 ± 0.01 | 0.16 ± 0.01 | <.0001 | 0.35 ± 0.01 | 0.26 ± 0.01 | 0.15 ± 0.01 | <.0001 | 0.39 ± 0.01 | 0.24 ± 0.01 | 0.13 ± 0.01 | <.0001 |
| Fish | 1.23 ± 0.02 | 1.15 ± 0.02 | 0.99 ± 0.02 | <.0001 | 1.37 ± 0.02 | 1.09 ± 0.02 | 0.95 ± 0.02 | <.0001 | 1.61 ± 0.02 | 1.11 ± 0.02 | 0.64 ± 0.02 | <.0001 |
| Meat | 0.62 ± 0.01 | 0.46 ± 0.01 | 0.34 ± 0.01 | <.0001 | 0.63 ± 0.01 | 0.46 ± 0.01 | 0.32 ± 0.01 | <.0001 | 0.61 ± 0.01 | 0.47 ± 0.01 | 0.32 ± 0.01 | <.0001 |
| Miscellaneous animal foods | 0.07 ± 0.003 | 0.04 ± 0.003 | 0.02 ± 0.003 | <.0001 | 0.07 ± 0.003 | 0.04 ± 0.003 | 0.02 ± 0.003 | <.0001 | 0.07 ± 0.003 | 0.04 ± 0.003 | 0.02 ± 0.003 | <.0001 |

Abbreviations: Q, quintile.

^1^ All food intake values were adjusted for total energy intake using the residual method and expressed as servings/d. Estimated mean and standard error were obtained using the linear regression model after adjusting for age and sex.

^2^ *P* for linear trend was determined by treating the median value of each group as a continuous variable in the linear regression model.
