# Supplementary material for: Positive association of unhealthy plant-based diets with the incidence of abdominal obesity in Korea: a comparison of baseline, most recent, and cumulative average diets
Source: Epidemiol Health. 2022 Aug 2;44:e2022063. doi: 10.4178/epih.e2022063 (PMC9754918; doi:10.4178/epih.e2022063)
Supplement: Supplementary Material 8 — Hazard ratios (HRs) and 95% confidence intervals (CIs) for abdominal obesity according to the plant-based diet indices with salted vegetables categorized into the healthy plant food group (n=6054) [file epih-44-e2022063-suppl8.docx]

**Online supplementary material**

This appendix is a part of the original submission and has been peer reviewed.

Supplement to: Jung S and Park S. Positive association of unhealthy plant-based diets with the incidence of abdominal obesity: a comparison of baseline, most recent, and cumulative average diets

**Supplementary Material 1. Participant flow chart**

**Supplementary Material 2. Food items listed in the FFQ with 103 items and the FFQ with 106 items in the KoGES_Ansan Ansung Study**

**Supplementary Material 3. Illustration of the three approaches for analyzing repeated dietary measurements in the KoGES Ansan and Ansung Study**

**Supplementary Material 4. Food items constituting the 17 food groups using the KoGES_Ansan Ansung Study**

**Supplementary Material 5. Age- and sex-adjusted nutritional characteristics of the study participants according to 3 different plant-based diet indices (n=6054)**

**Supplementary Material 6. Age- and sex-adjusted food group intakes of the study participants according to 3 different plant-based diet indices (n=6054)**

**Supplementary Material 7. Adjusted HRs and 95% CIs for incident abdominal obesity according to the continuous uPDIs using restricted cubic splines**

**Supplementary Material 8. Hazard ratios (HRs) and 95% confidence intervals (CIs) for abdominal obesity according to the plant-based diet indices with salted vegetables categorized into the healthy plant food group (n=6054)**

**Supplementary Material 9. Hazard ratios (HRs) and 95% confidence intervals (CIs) for abdominal obesity according to plant-based diet indices after excluding incident cases of abdominal obesity occurring within the first two follow-up years (n=5538)**

**Supplementary Material 10. Hazard ratios (HRs) and 95% confidence intervals (CIs) for abdominal obesity according to the plant-based diet indices after excluding incident cases of hypertension, T2DM, dyslipidemia, and general obesity occurring before the development of abdominal obesity during the follow-up (n=5656)**

**Supplementary Material 8. Hazard ratios (HRs) and 95% confidence intervals (CIs) for abdominal obesity according to the plant-based diet indices with salted vegetables categorized into the healthy plant food group (n=6,054)**

|  | **Baseline diet only** | **Most recent diet** | **Cumulative average** |
| --- | --- | --- | --- |
|  | **Multivariable-adjusted HR (95% CI)^2^** | **Multivariable-adjusted HR (95% CI)^2^** | **Multivariable-adjusted HR (95% CI)^2^** |
| **hPDI** |  |  |  |
| Q1 | 1.00 (reference) | 1.00 (reference) | 1.00 (reference) |
| Q2 | 1.05 (0.90, 1.22) | 0.76 (0.65, 0.88) | 0.78 (0.67, 0.91) |
| Q3 | 0.99 (0.85, 1.16) | 0.86 (0.73, 1.00) | 0.76 (0.65, 0.88) |
| Q4 | 1.08 (0.93, 1.25) | 0.91 (0.78, 1.05) | 0.92 (0.79, 1.06) |
| Q5 | 1.12 (0.96, 1.30) | 0.90 (0.78, 1.04) | 0.95 (0.82, 1.10) |
| *P* for trend^1^ | 0.15 | 0.76 | 0.72 |
| **uPDI** |  |  |  |
| Q1 | 1.00 (reference) | 1.00 (reference) | 1.00 (reference) |
| Q2 | 1.13 (0.97, 1.31) | 1.10 (0.94, 1.28) | 0.92 (0.79, 1.08) |
| Q3 | 1.21 (1.03, 1.42) | 1.21 (1.03, 1.43) | 1.03 (0.88, 1.20) |
| Q4 | 1.29 (1.11, 1.51) | 1.38 (1.19, 1.60) | 1.13 (0.97, 1.32) |
| Q5 | 1.70 (1.46, 1.97) | 1.46 (1.24, 1.71) | 1.59 (1.36, 1.85) |
| *P* for trend^1^ | <.0001 | <.0001 | <.0001 |

Abbreviations: HR, hazard ratio; CI, confidence interval; PDI, plant-based diet index; hPDI, healthy plant-based diet index; uPDI, unhealthy plant-based diet index.

^1^ *P* for trend was determined by treating the median value of each group as a continuous variable using a Cox proportional hazard model.

^2^ The multivariable-adjusted model was adjusted for age (years), sex (men or women), total energy intake (kcal/d), high school graduate (yes or no), physical activity level (METs), current smoking (yes or no), alcohol intake (g/d), and body mass index at baseline.
