# Supplementary material for: Positive association of unhealthy plant-based diets with the incidence of abdominal obesity in Korea: a comparison of baseline, most recent, and cumulative average diets
Source: Epidemiol Health. 2022 Aug 2;44:e2022063. doi: 10.4178/epih.e2022063 (PMC9754918; doi:10.4178/epih.e2022063)
Supplement: Supplementary Material 9 — Hazard ratios (HRs) and 95% confidence intervals (CIs) for abdominal obesity according to plant-based diet indices after excluding incident cases of abdominal obesity occurring within the first two follow-up years (n=5538) [file epih-44-e2022063-suppl9.docx]

**Online supplementary material**

This appendix is a part of the original submission and has been peer reviewed.

Supplement to: Jung S and Park S. Positive association of unhealthy plant-based diets with the incidence of abdominal obesity: a comparison of baseline, most recent, and cumulative average diets

**Supplementary Material 1. Participant flow chart**

**Supplementary Material 2. Food items listed in the FFQ with 103 items and the FFQ with 106 items in the KoGES_Ansan Ansung Study**

**Supplementary Material 3. Illustration of the three approaches for analyzing repeated dietary measurements in the KoGES Ansan and Ansung Study**

**Supplementary Material 4. Food items constituting the 17 food groups using the KoGES_Ansan Ansung Study**

**Supplementary Material 5. Age- and sex-adjusted nutritional characteristics of the study participants according to 3 different plant-based diet indices (n=6054)**

**Supplementary Material 6. Age- and sex-adjusted food group intakes of the study participants according to 3 different plant-based diet indices (n=6054)**

**Supplementary Material 7. Adjusted HRs and 95% CIs for incident abdominal obesity according to the continuous uPDIs using restricted cubic splines**

**Supplementary Material 8. Hazard ratios (HRs) and 95% confidence intervals (CIs) for abdominal obesity according to the plant-based diet indices with salted vegetables categorized into the healthy plant food group (n=6054)**

**Supplementary Material 9. Hazard ratios (HRs) and 95% confidence intervals (CIs) for abdominal obesity according to plant-based diet indices after excluding incident cases of abdominal obesity occurring within the first two follow-up years (n=5538)**

**Supplementary Material 10. Hazard ratios (HRs) and 95% confidence intervals (CIs) for abdominal obesity according to the plant-based diet indices after excluding incident cases of hypertension, T2DM, dyslipidemia, and general obesity occurring before the development of abdominal obesity during the follow-up (n=5656)**

**Supplementary Material 9. Hazard ratios (HRs) and 95% confidence intervals (CIs) for abdominal obesity according to plant-based diet indices after excluding incident cases of abdominal obesity occurring within the first two follow-up years (n=5,538)**

|  | **Baseline diet only** | **Most recent diet** | **Cumulative average** |
| --- | --- | --- | --- |
|  | **Multivariable-adjusted HR (95% CI)^2^** | **Multivariable-adjusted HR (95% CI)^2^** | **Multivariable-adjusted HR (95% CI)^2^** |
| **PDI** |  |  |  |
| Q1 | 1.00 (reference) | 1.00 (reference) | 1.00 (reference) |
| Q2 | 1.12 (0.93, 1.33) | 0.93 (0.78, 1.11) | 0.74 (0.62, 0.88) |
| Q3 | 0.92 (0.77, 1.10) | 0.89 (0.75, 1.06) | 0.78 (0.66, 0.91) |
| Q4 | 0.87 (0.72, 1.04) | 0.75 (0.63, 0.90) | 0.62 (0.51, 0.74) |
| Q5 | 1.06 (0.89, 1.26) | 0.90 (0.75, 1.07) | 0.90 (0.76, 1.06) |
| *P* for trend^1^ | 0.66 | 0.04 | 0.13 |
| **hPDI** |  |  |  |
| Q1 | 1.00 (reference) | 1.00 (reference) | 1.00 (reference) |
| Q2 | 1.02 (0.86, 1.22) | 0.78 (0.65, 0.92) | 0.79 (0.66, 0.94) |
| Q3 | 0.98 (0.81, 1.17) | 0.74 (0.62, 0.88) | 0.80 (0.67, 0.95) |
| Q4 | 1.14 (0.96, 1.36) | 0.80 (0.68, 0.95) | 0.81 (0.68, 0.97) |
| Q5 | 1.01 (0.84, 1.21) | 0.91 (0.76, 1.09) | 0.90 (0.75, 1.08) |
| *P* for trend^1^ | 0.81 | 0.004 | 0.40 |
| **uPDI** |  |  |  |
| Q1 | 1.00 (reference) | 1.00 (reference) | 1.00 (reference) |
| Q2 | 1.20 (1.01, 1.44) | 1.11 (0.93, 1.32) | 1.13 (0.94, 1.35) |
| Q3 | 1.33 (1.11, 1.60) | 1.17 (0.97, 1.41) | 1.13 (0.94, 1.36) |
| Q4 | 1.30 (1.08, 1.56) | 1.34 (1.12, 1.60) | 1.14 (0.95, 1.37) |
| Q5 | 1.60 (1.33, 1.92) | 1.47 (1.22, 1.77) | 1.70 (1.42, 2.05) |
| *P* for trend^1^ | <.0001 | <.0001 | <.0001 |

Abbreviations: HR, hazard ratio; CI, confidence interval; PDI, plant-based diet index; hPDI, healthy plant-based diet index; uPDI, unhealthy plant-based diet index.

^1^ *P* for trend was determined by treating the median value of each group as a continuous variable using a Cox proportional hazard model.

^2^ The multivariable-adjusted model was adjusted for age (years), sex (men or women), total energy intake (kcal/d), high school graduate (yes or no), physical activity level (METs), current smoking (yes or no), alcohol intake (g/d), and body mass index at baseline.
