# Supplementary material for: Positive association of unhealthy plant-based diets with the incidence of abdominal obesity in Korea: a comparison of baseline, most recent, and cumulative average diets
Source: Epidemiol Health. 2022 Aug 2;44:e2022063. doi: 10.4178/epih.e2022063 (PMC9754918; doi:10.4178/epih.e2022063)
Supplement: Supplementary Material 10 — Hazard ratios (HRs) and 95% confidence intervals (CIs) for abdominal obesity according to the plant-based diet indices after excluding incident cases of hypertension, T2DM, dyslipidemia, and general obesity occurring before the development of abdominal obesity during the follow-up (n=5656) [file epih-44-e2022063-suppl10.docx]

**Online supplementary material**

This appendix is a part of the original submission and has been peer reviewed.

Supplement to: Jung S and Park S. Positive association of unhealthy plant-based diets with the incidence of abdominal obesity: a comparison of baseline, most recent, and cumulative average diets

**Supplementary Material 1. Participant flow chart**

**Supplementary Material 2. Food items listed in the FFQ with 103 items and the FFQ with 106 items in the KoGES_Ansan Ansung Study**

**Supplementary Material 3. Illustration of the three approaches for analyzing repeated dietary measurements in the KoGES Ansan and Ansung Study**

**Supplementary Material 4. Food items constituting the 17 food groups using the KoGES_Ansan Ansung Study**

**Supplementary Material 5. Age- and sex-adjusted nutritional characteristics of the study participants according to 3 different plant-based diet indices (n=6054)**

**Supplementary Material 6. Age- and sex-adjusted food group intakes of the study participants according to 3 different plant-based diet indices (n=6054)**

**Supplementary Material 7. Adjusted HRs and 95% CIs for incident abdominal obesity according to the continuous uPDIs using restricted cubic splines**

**Supplementary Material 8. Hazard ratios (HRs) and 95% confidence intervals (CIs) for abdominal obesity according to the plant-based diet indices with salted vegetables categorized into the healthy plant food group (n=6054)**

**Supplementary Material 9. Hazard ratios (HRs) and 95% confidence intervals (CIs) for abdominal obesity according to plant-based diet indices after excluding incident cases of abdominal obesity occurring within the first two follow-up years (n=5538)**

**Supplementary Material 10. Hazard ratios (HRs) and 95% confidence intervals (CIs) for abdominal obesity according to the plant-based diet indices after excluding incident cases of hypertension, T2DM, dyslipidemia, and general obesity occurring before the development of abdominal obesity during the follow-up (n=5656)**

**Supplementary Material 10. Hazard ratios (HRs) and 95% confidence intervals (CIs) for abdominal obesity according to the plant-based diet indices after excluding incident cases of hypertension, T2DM, dyslipidemia, and general obesity occurring before the development of abdominal obesity during the follow-up (n=5,656)**

|  | **Baseline diet only** | **Most recent diet** | **Cumulative average** |
| --- | --- | --- | --- |
|  | **Multivariable-adjusted HR (95% CI)^2^** | **Multivariable-adjusted HR (95% CI)^2^** | **Multivariable-adjusted HR (95% CI)^2^** |
| **PDI** |  |  |  |
| Q1 | 1.00 (reference) | 1.00 (reference) | 1.00 (reference) |
| Q2 | 1.15 (0.97, 1.37) | 1.03 (0.87, 1.21) | 0.75 (0.63, 0.88) |
| Q3 | 0.91 (0.76, 1.08) | 0.94 (0.80, 1.11) | 0.77 (0.66, 0.91) |
| Q4 | 0.95 (0.80, 1.13) | 0.84 (0.71, 0.99) | 0.61 (0.51, 0.73) |
| Q5 | 1.08 (0.91, 1.28) | 0.96 (0.81, 1.14) | 0.96 (0.82, 1.12) |
| *P* for trend^1^ | 0.98 | 0.17 | 0.44 |
| **hPDI** |  |  |  |
| Q1 | 1.00 (reference) | 1.00 (reference) | 1.00 (reference) |
| Q2 | 1.10 (0.92, 1.32) | 0.85 (0.71, 1.01) | 0.70 (0.58, 0.83) |
| Q3 | 1.12 (0.94, 1.34) | 0.80 (0.68, 0.96) | 0.83 (0.70, 0.98) |
| Q4 | 1.27 (1.07, 1.51) | 0.85 (0.72, 1.01) | 0.80 (0.68, 0.95) |
| Q5 | 1.14 (0.96, 1.35) | 0.97 (0.82, 1.15) | 0.94 (0.80, 1.11) |
| *P* for trend^1^ | 0.05 | 0.89 | 0.81 |
| **uPDI** |  |  |  |
| Q1 | 1.00 (reference) | 1.00 (reference) | 1.00 (reference) |
| Q2 | 1.19 (0.999, 1.43) | 1.02 (0.85, 1.21) | 1.02 (0.86, 1.22) |
| Q3 | 1.36 (1.14, 1.63) | 1.20 (1.00, 1.44) | 1.10 (0.92, 1.31) |
| Q4 | 1.32 (1.10, 1.57) | 1.29 (1.09, 1.53) | 1.10 (0.92, 1.31) |
| Q5 | 1.77 (1.49, 2.10) | 1.53 (1.28, 1.83) | 1.79 (1.50, 2.12) |
| *P* for trend^1^ | <.0001 | <.0001 | <.0001 |

Abbreviations: HR, hazard ratio; CI, confidence interval; PDI, plant-based diet index; hPDI, healthy plant-based diet index; uPDI, unhealthy plant-based diet index.

^1^ *P* for trend was determined by treating the median value of each group as a continuous variable using a Cox proportional hazard model.

^2^ The multivariable-adjusted model was adjusted for age (years), sex (men or women), total energy intake (kcal/d), high school graduate (yes or no), physical activity level (METs), current smoking (yes or no), alcohol intake (g/d), and body mass index at baseline.
